# Supplementary material for: Tuning valley polarization of moiré trapped biexcitons by fine structure occupation in WS2/WSe2 heterostructures
Source: Nat Commun. 2025 Dec 26;17:1089. doi: 10.1038/s41467-025-67846-7 (PMC12852937; doi:10.1038/s41467-025-67846-7)
Supplement: Supplementary file 1 — Supplementary Infomation [file 41467_2025_67846_MOESM1_ESM.pdf]

Tuning Valley Polarization of Moiré Trapped Biexcitons by Fine Structure Occupation in WS_2/WSe_2 heterostructures

Authors:

Yufei Jiang[§], Yongzhi She[§], Xinke Cheng, Qinghai Tan, Jinlong Yang, Yilong Zhao, Peiwu Liu, Min Wu, Xiaotian Dai, Zengkai Wang, Hongbing Cai*, Nan Pan*, Xiaoping Wang

Supplementary information

Supplementary Section 1: Optical image and SHG of the device.

Supplementary Section 2: the intralayer exciton emission from the heterostructure and monolayer regions.

Supplementary Section 3: The IX and IXX emission peak positions blueshift with increasing excitation power.

Supplementary Section 4: Estimation of exciton density.

Supplementary Section 5: Other evidences of the biexciton.

Supplementary Section 6: Power-dependent PL intensities of the IX and IXX.

Supplementary Section 7: The dynamics of IXX and IX.

Supplementary Section 8: The formation pathways of IX and IXX through different layers.

Supplementary Section 9: The PL spectra at CW laser excitation.

Supplementary Section 10: The relaxation processes from IXX to IX.

Supplementary Section 11: The TRPL of the interlayer biexciton.

Supplementary Section 12: The TRPL of the interlayer single exciton.

Supplementary Section 13: The energy splitting of the IX and IXX.

Supplementary Section 14: The polarization and the energy splitting of the IXX as a function of the excitation power.

Supplementary Section 15: The temperature and power dependent exchange interaction.

Supplementary Section 16: The peak position of interlayer exciton at different temperatures.

Supplementary Section 17: The power-dependent polarization of the IX and IXX at 20 K, 50 K and 80 K.

Supplementary Section 18: The model for temperature-dependent polarization of the IXX.

Supplementary Section 19: The theoretical Calculation of the biexciton fine structure.

Fig. S1-23

Supplementary Section 1: Optical image and SHG of the device

The **Fig. S1a-b** shows the optical image and measured SHG signal of our sample, demonstrating that our sample is an H-shaped heterostructure with a twist angle of $59.8° \pm 0.2$ degrees.

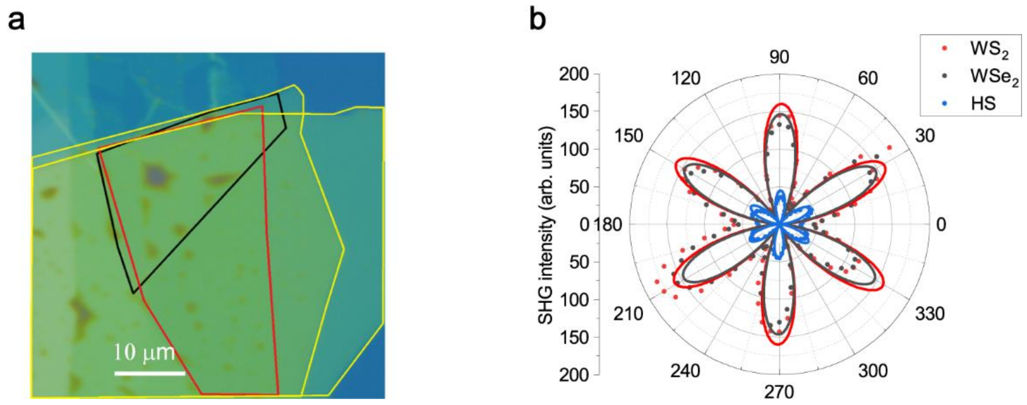

Fig. S1 Optical image and PL spectra of the device. a Optical microscope image of devices. The yellow box represents the top and bottom layers of hBN, the red box indicates WS_2, and the black box denotes WSe_2. **b** SHG signals of the WS_2, WSe_2 and heterostructure. This measurement confirms that the heterostructure is H-stacked with an interlayer twist angle of $59.8° \pm 0.2$ degrees.

Supplementary Section 2: the intralayer exciton emission from the heterostructure and respective monolayer regions.

Mechanically exfoliated TMDs often exhibit low defect concentrations (10^9 - 10^{10} cm^{-2}) and tend to be charge-neutral[1,2]. To qualitatively determine the doping of the samples, we measured the intralayer exciton photoluminescence (PL) spectrum of the monolayer regions as shown in the **Fig. S2a-b** (we use 680 nm and 532 nm lasers to excite WSe_2 and WS_2 monolayer regions, respectively). As shown in **Fig. S2a**, a peak at 2.01 eV dominates the monolayer WS_2 intralayer exciton emission, which accords with the neutral exciton energy commonly observed in other reports[3,4]. For monolayer WSe_2 intralayer exciton emission (**Fig. S2b**), there are two peaks. The higher-energy peak at 1.73 eV originates from WSe_2 neutral exciton[5]. The lower-energy one at 1.65 eV is associated with localized exciton[6]. No obvious charged exciton emission peak can be observed in these spectra. These results indicate that the investigated samples are at low

doping level.

We also measured the intralayer emission from the heterostructure region as shown in **Fig. S2c**, the results are well close to those measured in the monolayer regions. The WSe_2 intralayer exciton emission is still dominated by the localized exciton, and the WS_2 intralayer exciton emission shows strong neutral exciton peak at 2.01 eV and another weak peak around 1.92 eV, which may be related to the hybridized exciton[7].

Furthermore, FWHM of the interlayer and the intralayer excitons also indicate the high quality of our samples. Typically, the monolayer WS_2 and WSe_2 intralayer exciton emissions exhibit the FWHM ranging from a few meV to about a dozen meV[6,8]. Here the measured FWHM for the neutral exciton emissions in monolayer WS_2 and WSe_2 are 16 meV and 13 meV, respectively, well in line with the typical values.

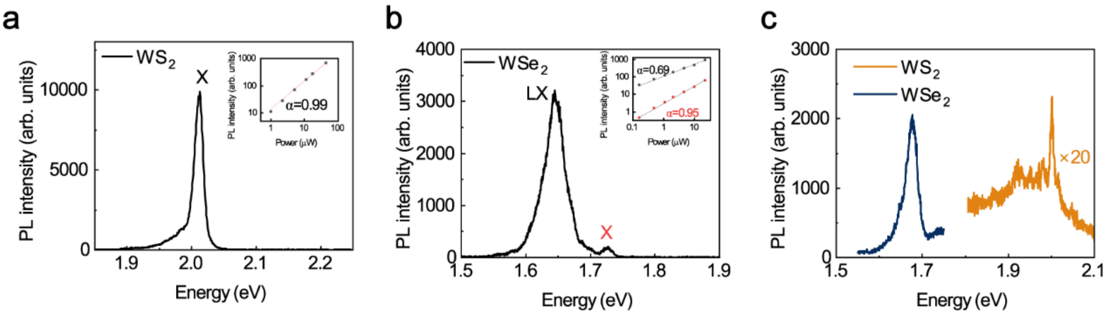

Fig. S2 Intralayer exciton luminescence in the monolayer and heterostructure regions. a The intralayer exciton photoluminescence spectrum of monolayer WS_2. It is dominated by the neutral exciton emission with an energy of 2.01 eV and a linewidth of 16 meV. The inset shows the power-dependent PL intensity of the neutral exciton emission in WS_2, the fitted power-law exponent is 0.99. **b** The intralayer exciton PL spectrum of monolayer WSe_2. It composes of a neutral exciton emission at 1.73 eV with the linewidth of 13 meV and a localized exciton emission at 1.65 eV with the linewidth of 36 meV. The fitted power-law exponents are 0.95 (1.73 eV) and 0.69 (1.65 eV), respectively, as shown in the inset. **c** The corresponding intralayer exciton emissions in the heterostructure regions.

Supplementary Section 3: The IX and IXX emission peak positions blueshift with increasing excitation power.

Due to dipole-dipole interactions, the emission peak positions of IX exhibit a blueshift as the exciton density increases, as shown in **Fig. S3**. The blueshift of IX is approximately $10\ meV$, which is consistent with other reports on hBN-encapsulated heterostructures, where a blueshift of around 10 meV has also been observed[2,9].

Additionally, a blueshift in the IXX peak position was observed with increasing excitation power. This behavior is attributed to the strong inherent dipole-dipole repulsion among IXX, which keeps the IXX energy higher than that of IX and maintains a blueshift trend consistent with that of IX. Moreover, the energy difference between IX and IXX is approximately 30 meV, in close agreement with the reported dipole–dipole repulsion U between two excitons trapped by a moiré potential [2].

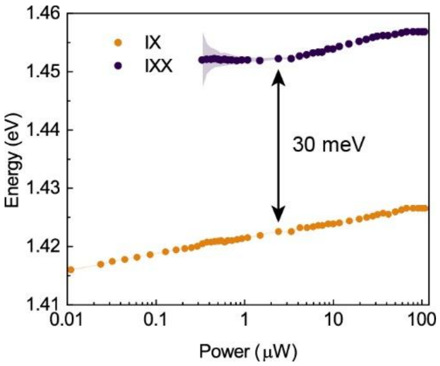

Fig. S3 The emission peak positions the IX and the IXX respectively as a function of the power. As the power increases, the IX and IXX emission peaks blueshift. The energy difference between IX and IXX maintain approximately 30 meV. The error bars represent the energy uncertainty obtained from the double-Voigt fitting of the PL spectrum.

Supplementary Section 4: Estimation of exciton density

We use the rate equation and lifetime to estimate the exciton density. For a laser energy of 1.68 eV, each photo carries the energy of 2.7×10^{-19} J. If considering the power intensity of $0.2\ \mu W$ and spot diameter of $1\ \mu m$, photons of $7.4 \times 10^{19}\ s^{-1}\ cm^{-2}$ will incident on the heterostructure. Using the absorptance by the heterostructure of 2% and a unitary conversion efficiency from intralayer to interlayer

exciton, we obtain the exciton generation rate $g = 1.5 \times 10^{18}\ \mathrm{s}^{-1}\ \mathrm{cm}^{-2}$. By fitting time-resolved PL intensity (**Fig. S4**), the exciton lifetime is around $\tau = 998$ ns. if we don't consider nonradiative recombination such as Auger recombination, the exciton density can be estimated approximately by $n = g\tau = 1.5 \times 10^{12}\ \mathrm{cm}^{-2}$. The value is very close to the moiré density of $1.8 \times 10^{12}\ \mathrm{cm}^{-2}$.

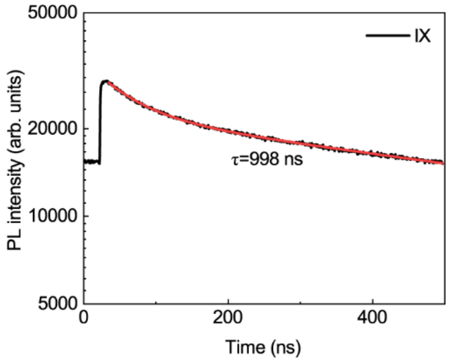

Fig. S4 The time-resolved photoluminescence (TRPL) of IX. Due to the relatively long exciton lifetime, a repetition frequency of 2 MHz is insufficient to capture the complete decay profile of the lifetime.

Supplementary Section 5: Other evidences of the biexciton

In this section, we discuss additional evidence for IXX as the biexciton. To rule out the involvement of other moiré states, we examine Zeeman splitting using linearly polarized excitation and circularly polarized detection (**Fig. S5**). The Zeeman splitting is fitted according to $\Delta E = g\mu_{\mathrm{B}}B$, where g is the g-factor and μ_{B} is the Bohr magneton. We get the g-factors of $+15.0$ for the exciton (IX) and $+13.7$ for the biexciton (IXX), respectively. This rules out the possibility of spin-triplet and spin-singlet interlayer exciton, as the difference between their g factors is 4[10]. Based on the negative polarization and the g-factor near 14, we conclude that both IX and IXX are exciton emissions located in the H_h^h minima and exciton in $-\mathrm{K}$ $(+\mathrm{K})$ valley emit $\sigma^+(\sigma^-)$ light[11].

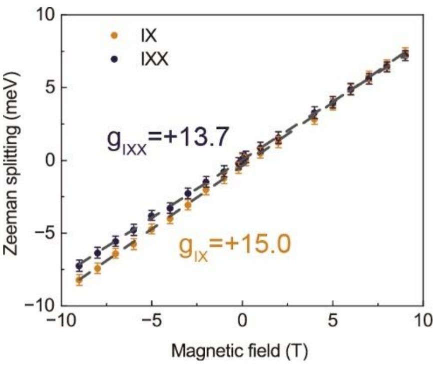

Fig. S5 Zeeman splitting of IX and IXX as a function of magnetic field. The purple and yellow dots represent the fitted Zeeman splitting values. Error bars are derived from the fitting uncertainty of the energy of two circularly polarized emissions.

Next, we rule out the possibility of trions. Mechanically exfoliated TMDs often exhibit low defect concentrations (10^9 - 10^{10} cm^{-2}) and tend to be charge-neutral. As shown in **Fig. S2**, we have measured the intralayer exciton PL of the monolayer WS_2 (**Fig. S2a**), WSe_2 (**Fig. S2b**) and the heterostructure (**Fig. S2c**). The fact that the intralayer exciton PL spectrum of monolayer regions is dominated by the neutral exciton with the absence of observable charged exciton emission. Furthermore, the intralayer exciton emission from the heterostructure region is similar to that of the monolayer region, with no obvious charged exciton emission is observed. This suggests that the monolayer and the heterostructure region in our sample is almost electrically neutral. Therefore, we ruled out the possibility of IXX emission being charged exciton luminescence.

We also rule out the possibility of quantum emitters. Previous studies have reported multiple narrow linewidth interlayer exciton emission peaks—with linewidths on the order of μeV—under low-power excitation in the nW range, suggesting their potential as quantum emitters[12]. These peaks merge into a broader emission peak at higher powers[13]. In our measurements, the interlayer exciton emission under low-power (nW) excitation exhibits a broad peak with a linewidth of approximately 15 meV centered near 1.42 eV. Moreover, we did not observe the characteristic peak merging behavior at high powers, thus excluding the possibility of quantum emission. The

emission characteristics we observed—including peak position and line shape—are also consistent with those recently reported for high-quality WS_2/WSe_2 heterostructures[14].

Supplementary Section 6: Power-dependent PL intensities of the IX and IXX

The full spectrum, with the excitation power spanning from 0.01 μW to 0.1 mW at 4 K, is provided in **Fig. S6a**. As illustrated, the IXX exhibits a superlinear rise at moderate powers. However, at high powers, the filling effect of moiré exciton becomes more significant, leading to the saturation of both IX and IXX intensities. Accordingly, we selected the PL intensities beyond but not much far away from the threshold power for fitting the power dependence. At this range, both a previous report[15] and our own double-peak fitting FWHM in **Fig. S6b** demonstrate that the FWHM of the IX reaches a minimum and remains rather stable, possibly due to the formation of correlated exciton states. Although the linewidth of IXX has a larger error at low powers near the threshold due to its weak intensity, using the peak height instead of the integrated intensity provides the most reliable measure to capture its characteristic superlinear nature.

Moreover, even if we consider the increased broadening of the IXX emission, such an effect would tend to increase the extracted power-law exponent by only about 0.2, which does not affect our conclusion that the biexciton power-law exponent is close to 2.

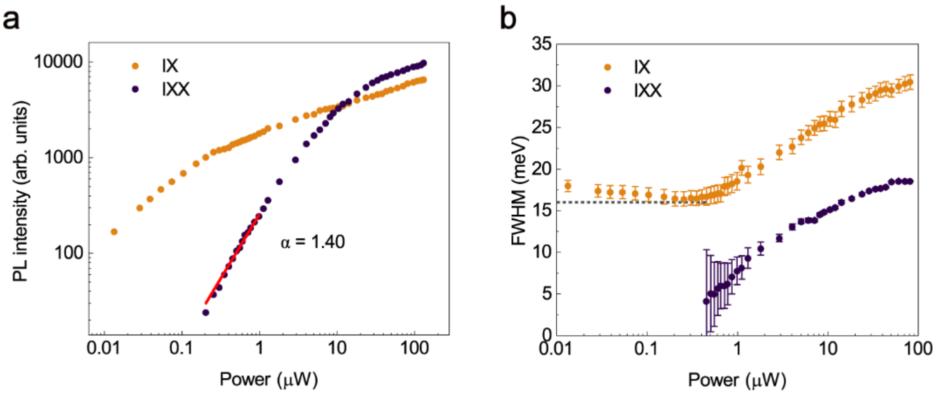

Fig. S6 The power dependence PL intensity and FWHM of IXX and IX emissions.
a The power dependence of IXX and IX PL intensities over a wide excitation power

range at 4 K. At low powers, IXX retains its characteristic of superlinear rise, exhibiting a power-law exponent of 1.4. At high powers, both IX and IXX exhibit saturation. **b** The power dependence FWHM of IX and IXX. For IX and IXX, the broadening changes primarily occur at high powers, while the broadening changes are minimal near the threshold power. The dashed line represents the minimum FWHM of interlayer singlets. The error bars represent the linewidth uncertainty obtained from the double-Voigt fitting of the PL spectrum.

Fig. S7 and **Fig. 1d** shows PL intensity of the IX and IXX at low powers under different temperatures. Fitting the data using a power-law model reveals that the intensity of IXX exhibits superlinear growth with increasing power. This behavior is consistent with the characteristics of biexcitons[14]. Furthermore, we could observe that power-law exponent increases with rising temperature. We attribute this behavior to the exciton delocalization effect from shallow non-radiative potential, which is might related to the defect sites in the sample. At low temperatures, a considerable portion of IX and IXX are trapped by the possibly existing non-radiative shallow potential wells, which hardly contributes to the luminescence and simultaneously suppresses the overall formation of IX and IXX, thereby reducing their power-law exponents[16]. As the temperature increases, these trapped IX and IXX are delocalized and more contributive to the luminescence, therefore leading to the increase toward the ideal values of 1 and 2 for IX and IXX, respectively.

Moreover, this delocalization mechanism may also explain why the IXX power-law exponent exceeds 2 at some temperatures. At high temperatures, when excitons are delocalized from shallow potential wells, it may become an additional generation channel for the IXX, thereby enhancing its emission beyond the conventional quadratic dependence.

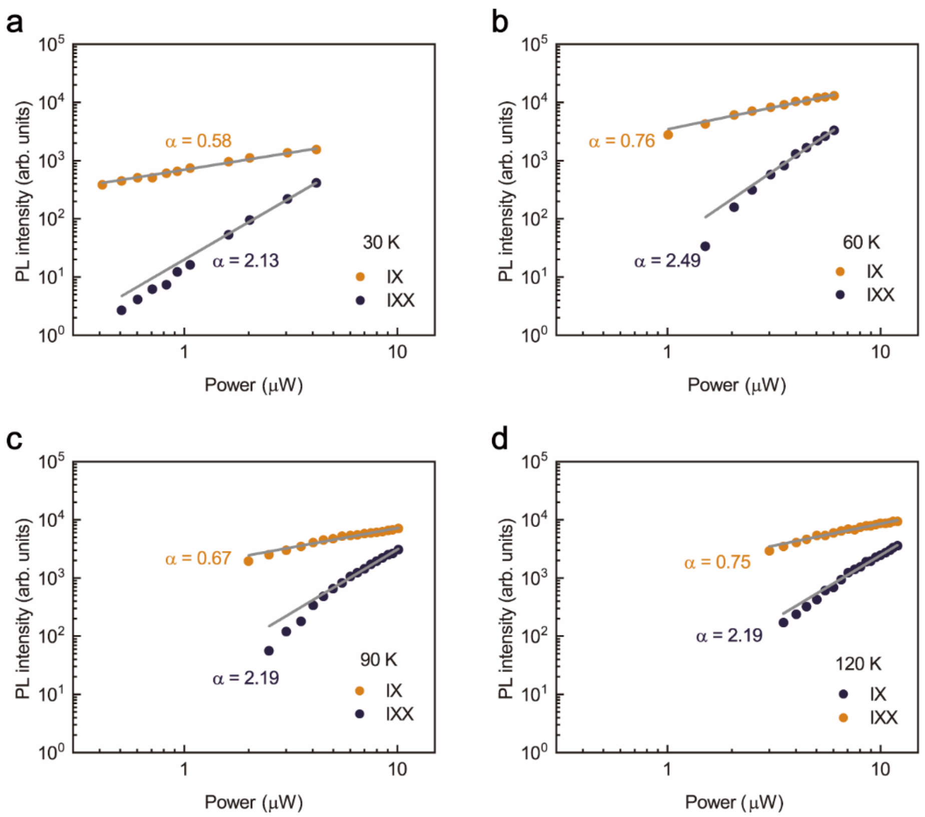

Fig. S7 PL intensity of the IX and IXX as a function of excitation power at 30 K (a), 60 K (b), 90 K (c) and 120 K (d).

Supplementary Section 7: The dynamics of IXX and IX.

In this section, we discuss dynamics of IX and IXX. Biexciton is of particular interest due to possible cascade emission. The **Fig. S8a, c** illustrates the three-level diagram of the ground state, interlayer singlet excitons, and interlayer biexcitons, where the high-energy biexciton state can relaxes to the IX state after one constituent exciton recombines. To better investigate the biexciton cascade emission, we simultaneously acquired time-resolved TRPL of IXX and IX under high- and low- power excitation at a repetition rate of 7.7 MHz. At low powers, biexcitons are absent, the IX dynamics reflect only its intrinsic relaxation (**Fig. S8b**). In contrast, at high powers, both IX and IXX are present. While IXX undergoes its own relaxation (**Fig. S8d**), this decay concomitantly leads to an increase in the IX population, manifesting as a rising component in the IX lifetime trace. Importantly, the decay time of IXX (to 1/e of its maximum) matches the rise time of IX to its maximum value—a clear signature of

biexciton cascade emission.

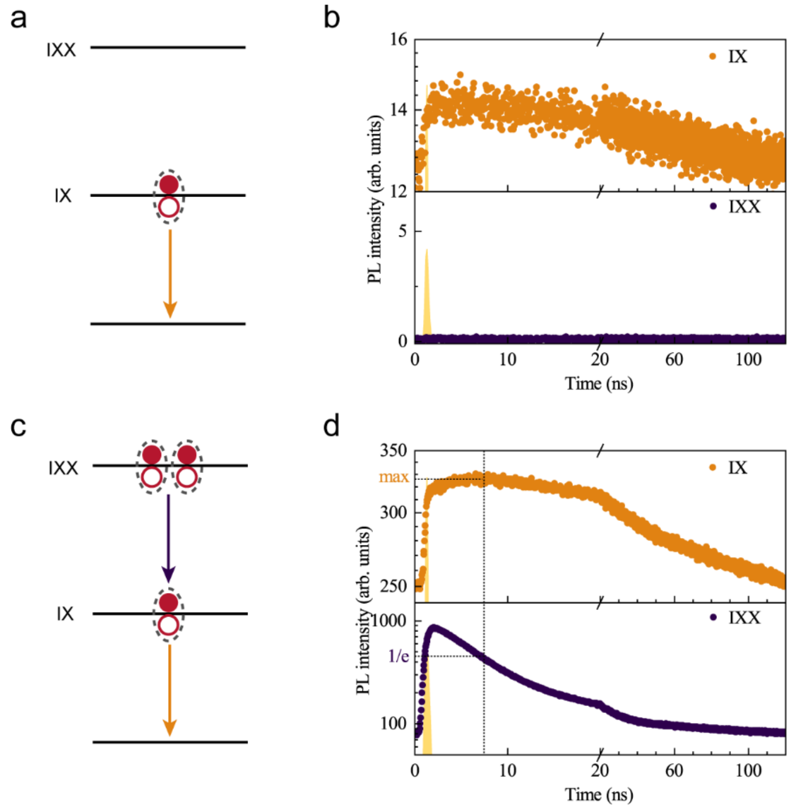

Fig. S8 Biexciton cascade emission. a Three-level energy diagram of biexciton, exciton, and ground state at low power, where biexcitons are absent. **b** Time-resolved photoluminescence at low power. the upper panel shows the time-resolved photoluminescence of IX, exhibiting only a decay process, while the lower panel shows the time-resolved photoluminescence of IXX (no emission). The yellow area represents the instrument response function. **c** Three-level energy diagram at high power. **d** Time-resolved photoluminescence at high power. the lower panel shows the time-resolved photoluminescence of IXX, and the upper panel shows the time-resolved photoluminescence of IX, including both injection and decay processes. The yellow area represents the instrument response function. The time for IXX relaxation to 1/e is the same as the time for IX to rise to its maximum value (as indicated by the dashed line).

Next, we investigate the dynamics of IX and IXX by measuring the power-dependent TRPL of IXX and IX. In **Fig. S9a**, we performed bi-exponential fit the IX

and found that the fast, slow, and average lifetimes remained unchanged (**Fig. S9b**). The average lifetime (1 μs) of IX is consistent with the observation in other report[2]. In sharp contrast, the IXX exhibits two orders of magnitude shorter lifetime of about 6 ns at the different powers (**Fig. S9c** and **Fig. S9d**). Recent report[17] show that the biexciton radiative recombination lifetime exceeds half of the single exciton lifetime ($\tau_{\mathrm{IXX}}/\tau_{\mathrm{IX}} > 0.5$), strongly indicating that the IXX's fast decay here does not originate from any radiative recombination process. Additionally, given the close distance of the two constituent excitons of the biexciton, *i.e.*, approximately 2 nm (as shown in **Fig. 4** in main text), exciton-exciton annihilation (EEA) is significantly enhanced. We speculate that EEA is the main reason for its short lifetime. The biexciton lifetime decreases slightly with increasing power, possibly due to enhanced EEA resulting from density changes.

Moreover, in the TMDs, the EEA rate can be described by $\gamma_{\mathrm{A}} = K_{\mathrm{A}} n_{\mathrm{eff}}$, where K_{A} is the EEA coefficient and n_{eff} is the local exciton density. The EEA rate of biexcitons can be estimated using the local exciton density: $\gamma_{\mathrm{A}} = \frac{2K_{\mathrm{A}}}{\pi r^2}$, where r is exciton radius of IXX. According to the report[18], the EEA coefficient of interlayer excitons is approximately $10^{-5} cm^2 s^{-1}$. Substituting $r = 2\ nm$, we can estimate the EEA rate to be approximately $1.6 \times 10^8 s^{-1}$. The rate corresponds to a lifetime of 6 ns, which is very close with our experimental results shown in Fig.S9d.

It is worth pointing out that the faster decay of IXX might also result from the enhanced non-radiative processes, because the strong dipolar repulsion between two excitons in the IXX can lead to its dissociation. However, considering that biexcitons can still exist at high temperatures of 120 K, we believe that the dissociation effect from dipolar repulsion is not large enough to account for the nearly two orders of magnitude reduction in the IXX lifetime. Therefore, our power-dependent lifetime measurements suggest that IX relaxation is dominated by its own recombination, while IXX relaxation is mainly driven by EEA. The detailed underlying mechanism still needs to be clarified theoretically.

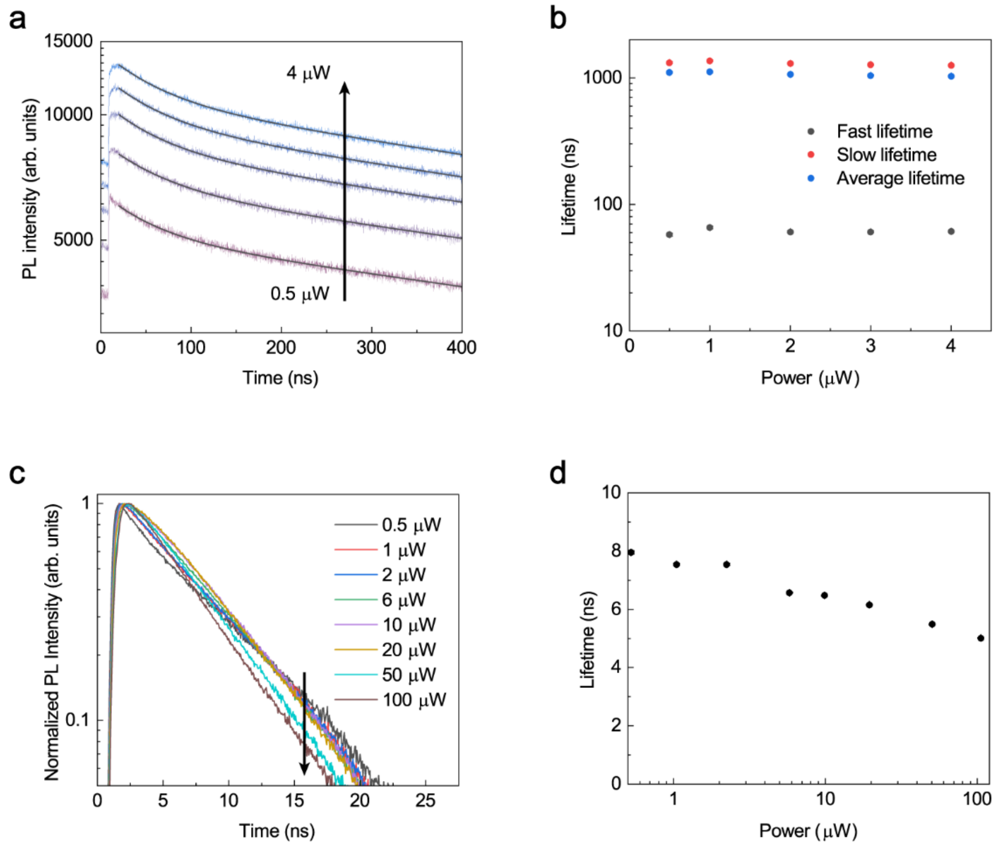

Fig. S9 The IX's and the IXX's lifetimes as a function of the excitation power. a The time-resolved photoluminescence of IX at different excitation powers, using repetition frequencies of 2 MHz. The black solid line represents the corresponding double exponential fit. **b** Fast, slow, and average lifetimes of the IX obtained from the fitting. **c** The normalized IXX's time-resolved photoluminescence at different excitation powers, using repetition frequencies of 38 MHz. Black arrows indicate a decrease in lifetime of the IXX. **d** IXX's lifetime as a function of excitation power. The lifetime is approximately 6 ns.

Consider the injection process from IXX to IX, we constructed a rate equation model to describe the IX's and IXX's dynamics[19]. Guiding by the schematic in **Fig. S10a**, we established the following rate equations:

$$\frac{dn_1}{dt} = -\frac{n_1}{\tau_{\mathrm{IX}}} - gSn_1 + \frac{n_2}{\tau_{\mathrm{IXX}}} + gSn_0$$

$$\frac{dn_2}{dt} = -\frac{n_2}{\tau_{\mathrm{IXX}}} + gSn_1$$

$$\frac{dn_0}{dt} = -gSn_0 + \frac{n_1}{\tau_{IX}} \quad \text{(S1)}$$

$$n_0 + n_1 + n_2 = n_{\mathrm{M}}$$

$$g = \alpha P$$

Here, n_0 and n_{M} represent the number of the empty and the total moiré potential wells within the irradiated area by the excitation light, respectively. n_1 and n_2 represent the number of single excitons and biexcitons, respectively (as sketched in **Fig. S10a**). τ_{IX} and τ_{IXX} are the lifetimes of IX and IXX, respectively. g is the exciton generation rate, where $\alpha \approx 7.5 \times 10^{18}$ s^{-1} cm^{-2} μW^{-2} (**Supplementary Section 4**), S is the area of a single unit cell of the moiré superlattice, and thus gS represents the rate of generating an exciton in single unit cell.

Considering that the lifetimes of IX and IXX do not change significantly with excitation power ($\tau_{\mathrm{IX}}{\sim}1$ μs and $\tau_{\mathrm{IXX}}{\sim}6$ ns), we investigate the I-P relationship of IX and IXX using the rate equations. The solution to the rate equation is:

$$n_1 = \frac{S\tau_{\mathrm{IX}} \cdot g}{1 + S\tau_{\mathrm{IX}} \cdot g + S^2\tau_{\mathrm{IX}}\tau_{\mathrm{IXX}} \cdot g^2} n_{\mathrm{M}} \quad \text{(S2)}$$

$$n_2 = \frac{S^2\tau_{\mathrm{IX}}\tau_{\mathrm{IXX}} \cdot g^2}{1 + S\tau_{\mathrm{IX}} \cdot g + S^2\tau_{\mathrm{IX}}\tau_{\mathrm{IXX}} \cdot g^2} n_{\mathrm{M}} \quad \text{(S3)}$$

Accordingly, we tried to fit the power dependence of the IX and the IXX relaxation using the functions $I_{\mathrm{IX}} = \frac{C_{\mathrm{IX}} \cdot P}{1+A \cdot P+B \cdot P^2}$ and $I_{\mathrm{IXX}} = \frac{C_{\mathrm{IXX}} \cdot P^2}{1+A \cdot P+B \cdot P^2}$. Where $A = S\tau_{\mathrm{IX}}\alpha$, $B = S^2\tau_{\mathrm{IX}}\tau_{\mathrm{IXX}}\alpha^2$, $C_{\mathrm{IX}} = S\tau_{\mathrm{IX}}\alpha n_{\mathrm{M}}$, and $C_{\mathrm{IXX}} = S^2\tau_{\mathrm{IX}}\tau_{\mathrm{IXX}}\alpha^2 n_{\mathrm{M}}$. As shown in the **Fig. S10b**, the fitting result does not match the IX data, although it matches the IXX data. From the expression of the solution, the order of the numerator (P) of n_1 is lower than that of the denominator (P^2). Therefore, n_1 is expected to decrease at high powers. Obviously, this does not conform to the experimental results. Therefore, some other factor that need to be considered is neglected in this physical picture.

Another report[9] indicates that interlayer excitons exhibit diffusion when their density is lower than the moiré density, whereas they are frozen and their diffusion is suppressed when exciton density exceeds the moiré density. Therefore, it is necessary to consider whether exciton diffusion affects the rate equation. Therefore, we revise the

above equation. When considering exciton diffusion, the number of $n_0 + n_1 + n_2$ is no longer equal to the number of moiré potential. We expand it to the first order of the exciton generation rate, resulting in $n_0 + n_1 + n_2 = n_{\mathrm{M}} + gn_{\mathrm{D}} + \delta(g^2)$, where n_{D} is the coefficient related to exciton diffusion. The solution to the equation is as follows:

$$n_1 = \frac{Sn_{\mathrm{D}}\tau_{\mathrm{IX}} \cdot g^2 + Sn_{\mathrm{M}}\tau_{\mathrm{IX}} \cdot g}{1 + S\tau_{\mathrm{IX}} \cdot g + S^2\tau_{\mathrm{IX}}\tau_{\mathrm{IXX}} \cdot g^2} \tag{S4}$$

$$n_2 = \frac{S^2\tau_{\mathrm{IX}}\tau_{\mathrm{IXX}}n_{\mathrm{D}} \cdot g^3 + S^2\tau_{\mathrm{IX}}\tau_{\mathrm{IXX}}n_{\mathrm{M}} \cdot g^2}{1 + S\tau_{\mathrm{IX}} \cdot g + S^2\tau_{\mathrm{IX}}\tau_{\mathrm{IXX}} \cdot g^2} \tag{S5}$$

Considering the exciton diffusion, the order of the numerator (P^2) and denominator (P^2) of n_1 are the same, and the IX intensity no longer decreases at high powers, which is more consistent with the experimental results. We use simplified functions $I_{IX} = \frac{C_{\mathrm{IX}}P^2 + D_{\mathrm{IX}}P}{1+AP+BP^2}$ and $I_{IXX} = \frac{C_{\mathrm{IXX}}P^3 + D_{\mathrm{IXX}}P^2}{1+AP+BP^2}$ to fit the power dependence of the IX and IXX intensities. The results are shown in the **Fig. S10c**, one can see that the equations now fit the experimental results fairly well. The fitting parameters are $A = 4.26\,\mu W^{-1}$, $B = 0.158\,\mu W^{-2}$, $C_{\mathrm{IX}} = 8749\,\mu W^{-2}$, $D_{\mathrm{IX}} = 1124\,\mu W^{-1}$, $C_{\mathrm{IXX}} = 0\,\mu W^{-3}$, $D_{\mathrm{IXX}} = 1816\,\mu W^{-2}$, respectively. The fitted ratio of $\tau_{\mathrm{IX}}/\tau_{\mathrm{IXX}} = A^2/B \sim 115$, close to the experimental counterpart $\tau_{\mathrm{IX}}/\tau_{\mathrm{IX}X} = \frac{1\,\mu s}{6\,ns} \sim 166$. The fitted area of a single unit cell of the moiré superlattice $S = \frac{A}{\alpha\tau_{\mathrm{IX}}} = 57\,nm^2$ also agrees well with the result estimated from the moiré period of 7.6 nm ($S \sim a_{\mathrm{M}}^2 = 58\,nm^2$). Furthermore, one can see that the fitted $\frac{C_{\mathrm{IX}}}{D_{\mathrm{IX}}} \gg \frac{C_{\mathrm{IXX}}}{D_{\mathrm{IXX}}} = 0$, contradicting with the expected equal result $n_{\mathrm{D}}/n_{\mathrm{M}}$ (related to diffusion). We attribute this discrepancy to the greatly different diffusion behaviors of the IX and the IXX: IX exhibits significant diffusion, while IXX shows almost no diffusion (n_{D} of IXX approaches 0).

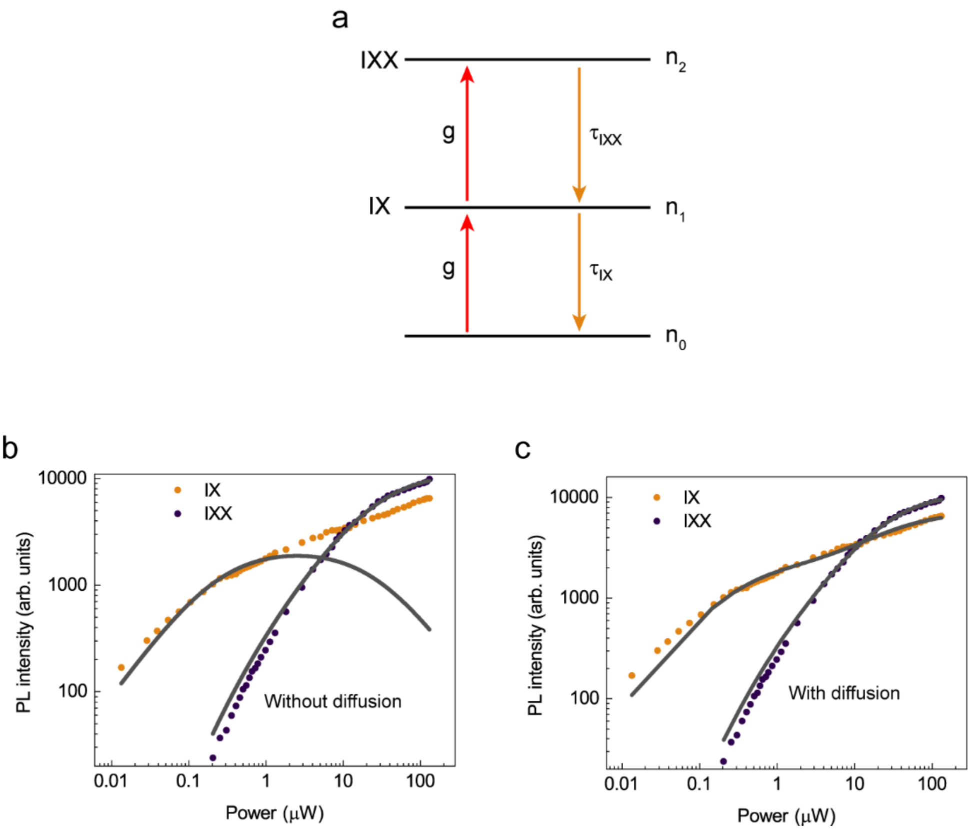

Fig. S10 Rate equation fitting without or with considering the diffusion. a Three-level model of IXX, IX, and empty moiré potential. **b** The rate equation fitting of the power-dependent PL intensity for the IX and the IXX without the diffusion. **c** The rate equation fitting with considering the exciton diffusion. The gray solid line represents the fitting result.

Supplementary Section 8: The formation pathways of IX and IXX through different layers.

Due to the Type-II band alignment in WS_2/WSe_2 heterostructure, as long as an intralayer exciton is excited in the $-K$ valley of WSe_2, the electron undergoes a rapid charge transfer process to the $+K$ valley of WS_2, forming an interlayer exciton with its electron in the WS_2 layer and its hole in the WSe_2 layer (we denote an interlayer exciton IX with a hole in the $-K$ $(+K)$ valley of WSe_2 and an electron in the $+K$ $(-K)$ valley of WS_2 as a $-K$ $(+K)$ valley interlayer exciton), as shown in **Fig. S11a**. Due to intervalley scattering, IX can remain in the $-K$ valley (**Fig. S11a**) or scatter into the $+K$ valley (**Fig. S11b**), leading to a distribution in both valleys even if only the $-K$

valley of WSe_2 is initially excited. This explains the observed weak valley polarization of the IX

When another exciton is excited into the same moiré potential (**Fig. S11c** and **Fig. S11d**), the two IXs can combine together to form an interlayer biexciton. Depending on the valley type of the constituent interlayer excitons, there are two types of interlayer biexcitons: 1. Under selective excitation of the $-\mathrm{K}$ valley, the $-\mathrm{K}$ valley exciton may combine with a $-\mathrm{K}$ exciton without intervalley scattering, as shown in **Fig. S11c**. Here, the moiré potential contains two combined interlayer excitons both in the –K valley. This forms an intravalley biexciton. 2. Alternatively, the $-\mathrm{K}$ valley exciton can also combine with an exciton of $+\mathrm{K}$ valley that undergo intervalley scattering, as shown in **Fig. S11d**. In this case, the moiré potential hosts two interlayer excitons occupying the $+\mathrm{K}$ and $-\mathrm{K}$ valleys, respectively. This forms an intervalley biexciton.

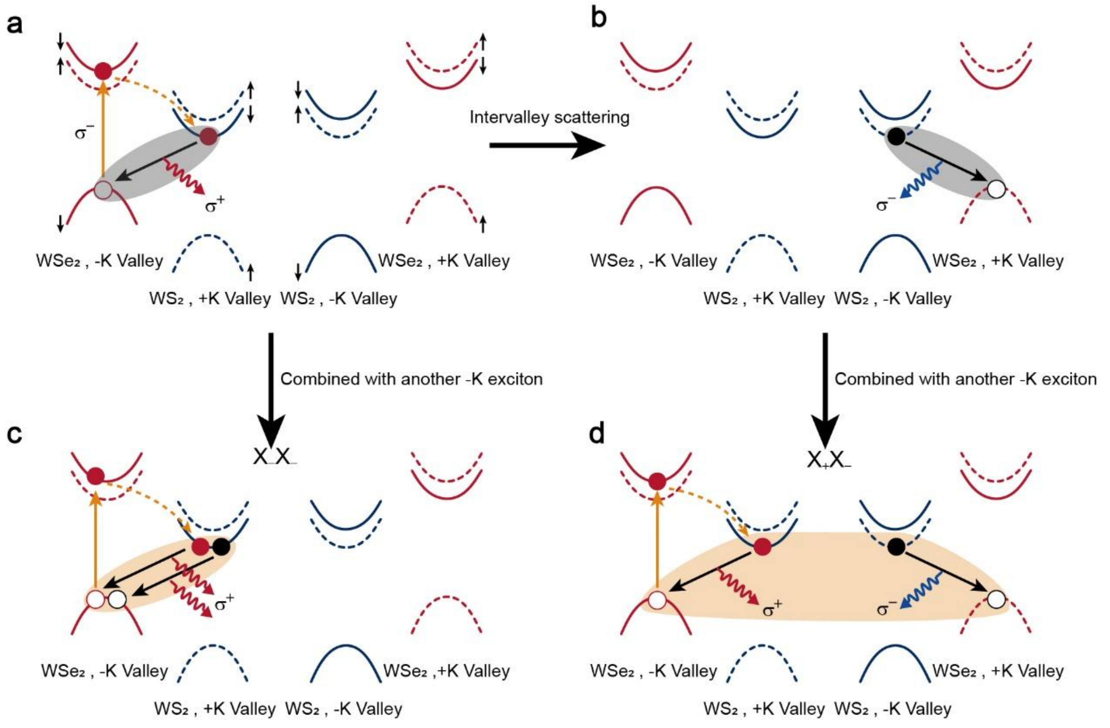

Fig. S11 Interlayer exciton formation processes related to layers. a Formation of interlayer single excitons. When the $-\mathrm{K}$ valley of WSe_2 is excited by σ^- circularly polarized light (yellow solid arrows), $-\mathrm{K}$ valley interlayer excitons are formed due to charge transfer (yellow dashed arrows), which emit σ^+ circularly polarized light. The black arrows indicate the spins of electrons and holes. **b** $-\mathrm{K}$ valley excitons undergo

intervalley scattering into the $+\mathrm{K}$ valley, which emit σ^{-} circularly polarized light. **c** Formation of intravalley biexcitons. Under selective excitation of the $-\mathrm{K}$ valley, the $-\mathrm{K}$ valley exciton can also combine with an exciton that remains in the $-\mathrm{K}$ valley without scattering (**a**). Here, the moiré potential contains two combined interlayer excitons both in the $-\mathrm{K}$ valley. Therefore, intravalley biexcitons is polarized emission. **d** Formation of intervalley biexcitons. Under selective excitation of the $-\mathrm{K}$ valley, the $-\mathrm{K}$ valley exciton may combine with a $-\mathrm{K}$ exciton that has undergone intervalley scattering (**b**). Here, the moiré potential hosts two interlayer excitons occupying the $+\mathrm{K}$ and $-\mathrm{K}$ valleys, respectively. Therefore, intervalley biexcitons is unpolarized emission.

Supplementary Section 9: The PL spectra at CW laser excitation

Fig. S12 show the PL spectra at CW laser excitation. We chose to collect the signal using single-mode fiber, yielding results similar to those in the main text, and allowing to observe the biexciton fine structure.

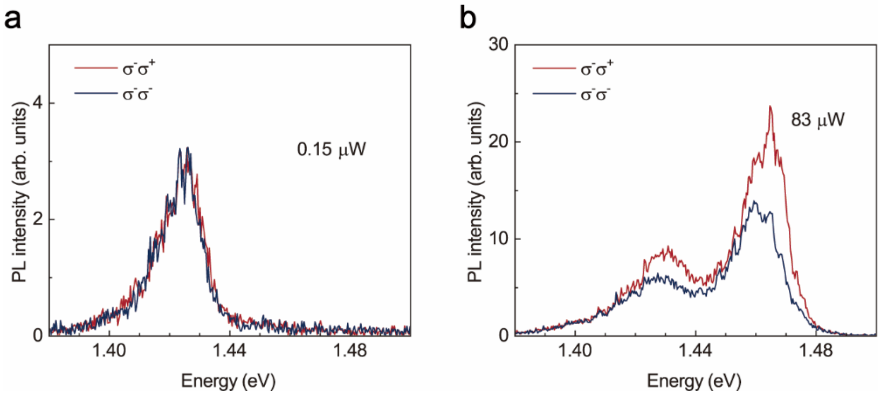

Fig. S12 Circular polarization-resolved interlayer exciton PL spectra at CW laser excitation. a and **b**, The PL spectra at 0.15 μW and 83 μW.

Supplementary Section 10: The relaxation processes from IXX to IX

In moiré-trapped systems, interlayer excitons may exhibit discrete excited-state energy levels, complicating the relaxation process[20]. However, according to other calculations[17], biexciton luminescence is primarily dominated by its transition to the

single exciton ground state, with only weak signals indicating relaxation to excited states. In our research, we find no indication of the IXX relaxation to excited states of the IX, a process that would produce a series of alternately polarized IXX peaks in the PL spectrum[17]. Emissions from excited IX states are also absent, which would have been visible as multiple alternately polarized IX peaks under low-power excitation[21]. This behavior matches other reports[2,14], where only IXX–to–ground-state-IX relaxation is detected, a process we illustrate in **Fig. S13**. For intravalley biexcitons X_-X_- (red harmonic oscillator well), relaxation to the ground state IX (yellow harmonic oscillator well) results in the emission of σ^+ circularly polarized light. For intervalley biexcitons X_+X_- (gray harmonic oscillator well), relaxation to the ground state IX results in the emission of either σ^+ or σ^- circularly polarized light.

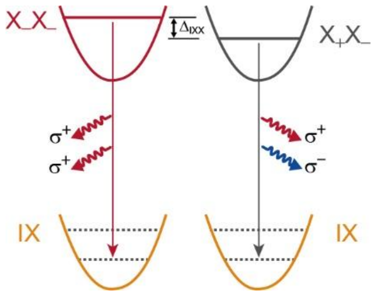

Fig. S13 The relaxation process of biexciton to single exciton. An intravalley biexciton emits circularly polarized light, while an intervalley biexciton emits unpolarized light. Furthermore, the relaxation of biexciton states is primarily dominated by their transitions to the ground state exciton, with no observable experimental evidence of relaxation to the exciton's excited states. The red, gray, and yellow harmonic oscillator wells represent the intravalley IXX, intervalley IXX, and IX, respectively. Δ_{IXX} represents the fine structure splitting of the IXX.

Supplementary Section 11: The TRPL of the interlayer biexciton

Fig. S14 illustrates the time-resolved photoluminescence measurements of the IXX. As the power is equal to P_{th} (**Fig. S14a**), σ^- and σ^+ circularly polarized luminescence maintains identical amplitude and dynamics. This is consistent with previous research, which suggests that valley configuration of biexciton is X_+X_-, and polarization of

biexciton remains zero. At higher powers, we observe that the amplitude of the σ^+ emission becomes higher than that of σ^- after laser pulse (**Fig. S14b**). This indicates the generation of intravalley biexcitons, as only intravalley biexcitons can emit circularly polarized light, as shown in **Fig. 1b**.

We then calculate the polarization at different powers by $DCP(t) = (I_{\sigma-}(t) - I_{\sigma+}(t))/(I_{\sigma-}(t) + I_{\sigma+}(t))$, shown in **Fig. S14c**. we fit polarization data with $DCP = P_0 e^{-\frac{t}{\tau_V}}$, P_0 is initial valley polarization and τ_V represents valley lifetime. As described in main text, the intravalley biexcitons can emit circularly polarized light, while intervalley biexcitons only emit unpolarized light. Therefore, if only intervalley biexcitons are present, P_0 should be close to 0. when intravalley biexciton emission occurs, P_0 should increase. we observe that P_0 increases rapidly with power (**Fig. S14d** and dotted arrow in **Fig. S14c**). Comparing the trend of P_0 with the IXX polarization (**Fig. 2b**), we observe that both exhibit similar trends as power increases. This indicates that the increase in valley polarization is primarily due to the increase of P_0, which suggests that more intravalley biexcitons (X_X_) are being excited as the power increases, thereby increasing the valley polarization.

In addition, by fitting $I_{\sigma-}(t) + I_{\sigma+}(t)$ by $I = I_0 e^{-\frac{t}{\tau}}$, we can obtain the IXX lifetime τ. **Fig. S14e** show the IXX lifetime and valley lifetime as a function of the excitation power. The ratio τ/τ_V remains approximately constant as power increase, as shown in **Fig. S14f**. Therefore, we rule out the possible cause of reduced intervalley scattering to the increase of valley polarization.

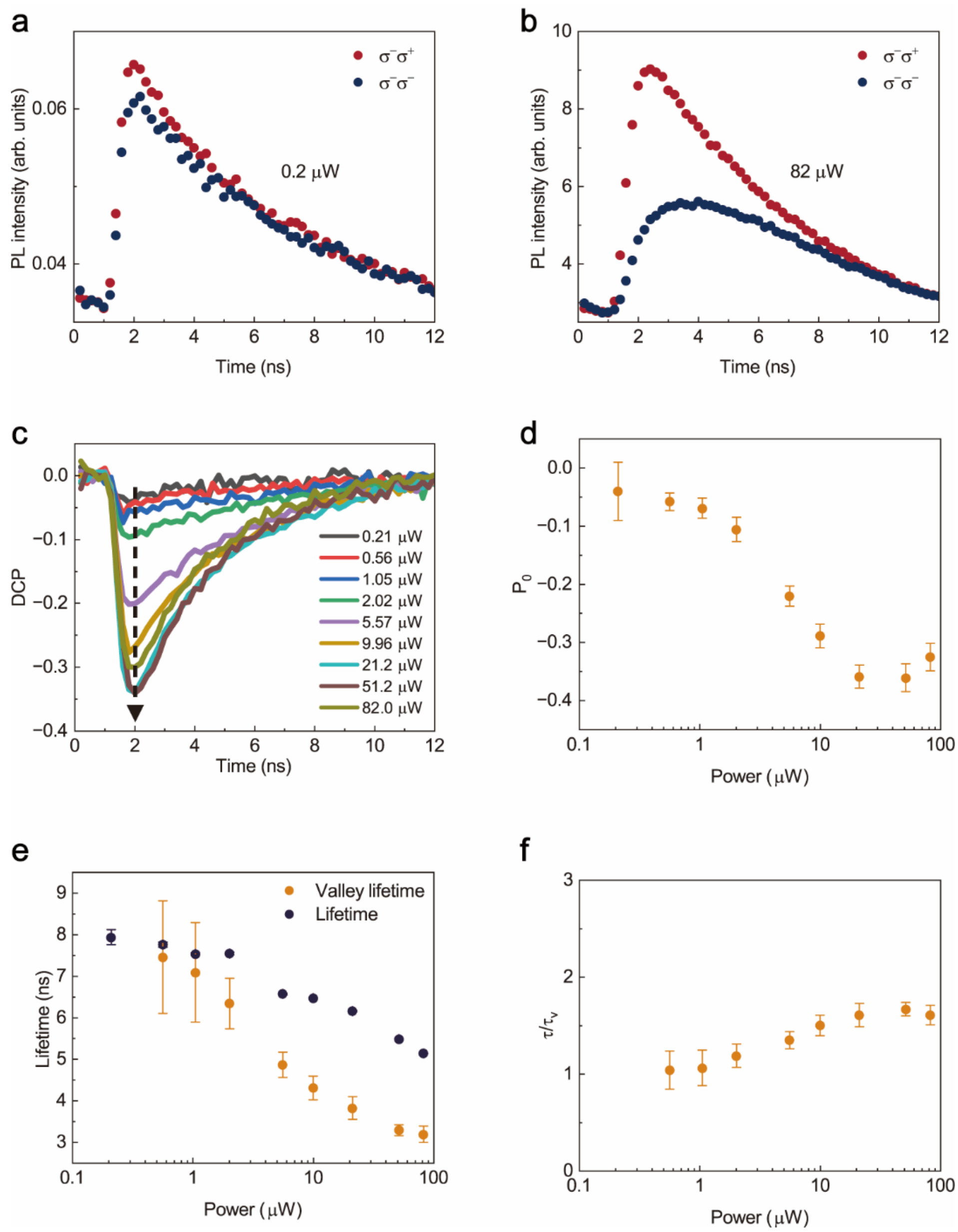

Fig. S14 The TRPL of the IXX. a, b Circularly polarized TRPL at excitation power of 0.2 μW and 82 μW. At 0.2 μW, the σ^- and σ^+ luminescence exhibit identical amplitude and dynamics, indicating the generation of the X_+X_-. At 82 μW, the amplitude of the σ^+ emission becomes larger than that of σ^-, which suggests the generation of X_-X_-. **c** Time-resolved polarization at different excitation powers. Dotted arrow represents increased initial valley polarization P_0. **d** The initial valley polarization P_0. The error bars represent the fitting uncertainty of the initial valley polarization. **e** The IXX lifetime and valley lifetime as a function of the excitation power. The error bars represent the time uncertainty. **f** The ratio τ/τ_v of exciton

lifetime to valley lifetime. It is nearly independent of power, therefore ruling out the possible cause of reduced intervalley scattering to the increase of valley polarization.

Supplementary Section 12: The TRPL of the interlayer single exciton

As shown in **Fig. 2a** and **Fig. 2b**, the polarization of IX increases with rising power. In this section, we discuss the underlying reasons for this behavior based on TRPL measurements.

At low excitation power (34 nW), when we excite the −K valley by σ^- circularly polarized laser, the PL intensity of IX in −K valley (σ^+) increases, while the change of +K valley (σ^-) is negligible, as shown in **Fig. S15a**. With time decay, the exciton undergoes intervalley scattering and recombination. To quantify the polarization dynamics, we calculate the polarization using the formula $DCP(t) = (I_{\sigma^-}(t) - I_{\sigma^+}(t))/(I_{\sigma^-}(t) + I_{\sigma^+}(t))$. The resulting data is shown in **Fig. S15c**. we fit the data with $DCP(t) = P_0 e^{-t/\tau_\mathrm{v}}$, where the valley lifetime $\tau_\mathrm{v} = 16.5$ ns.

However, under high excitation power (3.7 µW) with pulsed laser, we observe a suppression of PL intensity in the +K valley, whereas the −K valley exhibits enhanced PL emission (**Fig. S15b**). We also calculate the polarization and find the valley lifetime $\tau_\mathrm{v} = 14.1$ ns, as shown as **Fig. S15c**. By comparing the results at 34 nW and 3.7 µW, we conclude that valley lifetime alone cannot fully explain the increased polarization of the IX. Instead, the increasing polarization of IX is closely related to the emergence of the IXX. When the power slightly exceeds P_th, intervalley biexciton emerges. Consequently, the excited −K valley exciton combine with X_+ and generate $\mathrm{X}_+\mathrm{X}_-$ biexciton, leading to a decrease in the density of X_+ (shown in **Fig. S15d** highlight by black circle). The increased number of X_- may be attributed to excitation from laser edge, where the exciton density is lower than the moiré density. Both increased X_- and decreased X_+ result in enhanced polarization.

At power intensity approaches 10 µW, we observe a significant increase in the polarization of IXX, while the polarization of IX shows less of a change. This is likely because that the polarization of IXX origins from $\mathrm{X}_-\mathrm{X}_-$, which reduces the number of

X_- and weaken the regulation for IX in polarization.

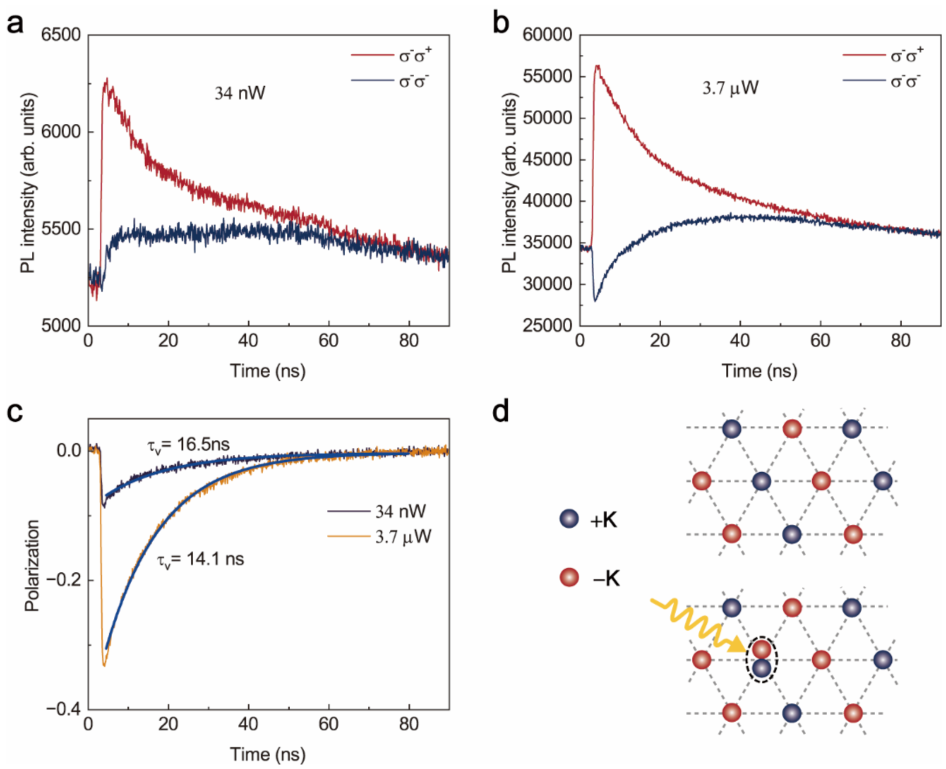

Fig. S15 The TRPL of the IX. a, b Circularly polarized TRPL at power intensity of 34 nW and 3.7 μW. At 3.7 μW, we observe that the PL intensity of σ^- exhibits a dip when the laser pulse arrives. **c** The time-resolved polarization of IX at 34 nW and 3.7 μW. **d** Schematic of interlayer exciton filling in moiré superlattices. Blue and red balls represent the excitons in the +K and −K valleys. At low excitation power (top), only interlayer single excitons are excited. As power increase, X_+X_- also forms by exciting an exciton of −K valley in the moiré site filled by +K valley exciton (bottom).

Supplementary Section 13: The energy splitting of the IX and IXX

We fit the peak positions at different powers and calculate the energy splitting using the following formula: $\Delta E\ (\sigma^{\pm}\ \text{exc.}) = E(\sigma^{\mp}) - E(\sigma^{\pm})$, $\sigma^{\pm}$ excitation represent the excitation helicities, and $E(\sigma^{\pm})$ is peak position of circularly polarized luminescence

of $\sigma^{\pm}$. The result show in **Fig. S16a**.

Fig. S16b shows the energy level diagram of IX and IXX. The model also considers the exchange interaction between moiré-trapped single excitons and the total exciton background. Since this interaction is valley-dependent, single excitons in the two valleys exhibit an energy difference Δ_{IX}. This interaction is taken into account in the biexciton fine structure. As a result, the energy difference in the biexciton fine structure consists of two components. One component is the difference due to the internal exchange interaction Δ_{IXX} within the biexciton, as discussed in the main text, which is independent of the total exciton density; the other component corresponds to the energy difference Δ_{IX} of the single exciton, which is related to the exciton density and valley distribution. At low excitation powers, biexciton luminescence is dominated by X_+X_-. Due to the valley splitting of IX, the peak energy splitting of IXX is similar to that of IX (**Fig. S16b**). Therefore, the peak energy splitting of IXX minus that of IX should be close to zero. At high excitation powers, the intravalley biexciton X_-X_- also contributes to the emission, and the splitting of IXX similarly includes that of IX. To extract the energy splitting of the biexciton more accurately, we subtract the energy splitting of the single excitons from that of the biexciton: $\Delta E_{\text{IXX}} = \Delta E(\text{IXX}) - \Delta E(\text{IX})$.

We observe that the valley splitting exhibits two distinct regimes: increasing and decreasing with increasing power. We will now discuss the potential origins of these two regimes.

In the initial stage under low excitation powers, the valley splitting of interlayer excitons increases with rising excitation powers. This behavior can be attributed to valley-dependent exchange interactions. Previous study have reported that when the exciton density in the +K valley ($n_{+\text{K}}$) exceeds that in the −K valley ($n_{-\text{K}}$), the exchange interaction raises the energy of the +K valley above that of the −K valley, thereby inducing valley splitting[22]. Moreover, the magnitude of this splitting increases with the population difference $\Delta n = n_{+\text{K}} - n_{-\text{K}}$, due to a strengthening exchange interaction. This mechanism aligns well with our observations: the increase in valley polarization of IX in this regime leads to a growing population difference, which in turn drives the

enhancement of the valley splitting.

The difference emerges in the second stage, where the IX energy splitting begins to decrease under higher excitation powers. While direct evidence explaining this decline is currently lacking, we speculate that it may be attributed to Coulomb screening. Theoretical studies suggest that Coulomb screening could play a significant role in exciton-exciton interactions[23]. As the exciton density rises, enhanced Coulomb screening may weaken the interactions between excitons, leading to a reduction in the IX splitting. We hypothesize that this mechanism may account for the observed decrease in splitting.

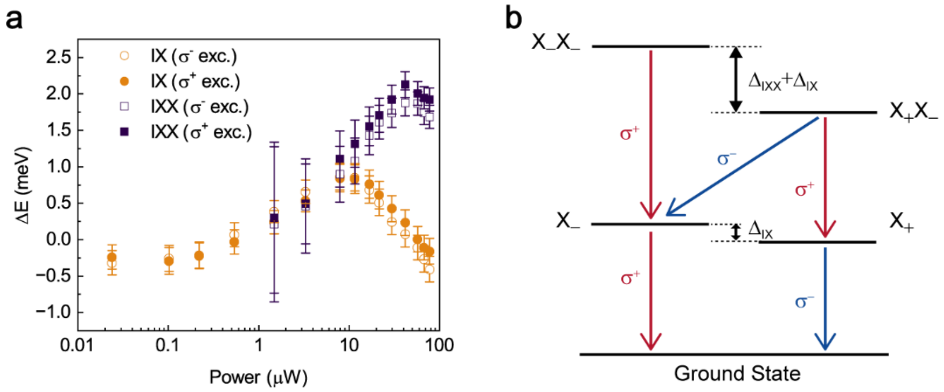

Fig. S16 The energy splitting of $\boldsymbol{\sigma^-}$ and $\boldsymbol{\sigma^+}$ excitation. a The energy splitting of both IX and IXX can be obtained by $\Delta E\ (\sigma^{\pm}\ \text{exc.}) = E(\sigma^{\mp}) - E(\sigma^{\pm})$, where $\sigma^{\pm}$ exc. represent the excitation helicities, and $E(\sigma^{\pm})$ correspond to the peak energies of IX or IXX. The error bars represent the energy uncertainty obtained from the double-Voigt fitting of the PL spectrum. **b** Energy level diagram of IX and IXX. Δ_{IX} represents the energy splitting of IX, caused by exciton-exciton exchange interaction. Δ_{IXX} is fine structure of biexcitons.

Supplementary Section 14: The polarization and the energy splitting of the IXX as a function of the excitation power

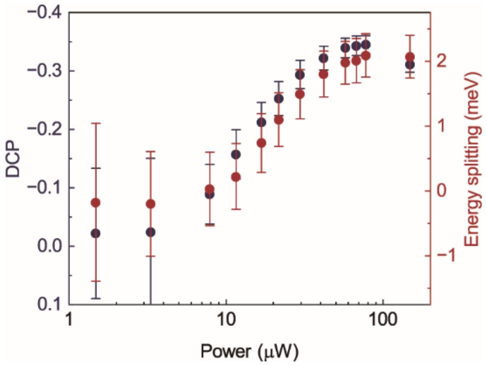

Fig. S17 The polarization and the energy splitting of the IXX as a function of the excitation power. Basically, the polarization and the energy splitting are positively correlated.

Supplementary Section 15: The temperature and power dependent exchange interaction.

It is well established that in TMD, the valley depolarization process was dominated by exchange interaction, which shortens the valley lifetime and consequently degrades the valley polarization. The strength of the exchange interaction depends primarily on the electron–hole wavefunction overlap[14], interlayer excitons exhibit significantly weaker exchange interaction compared to intralayer counterparts due to the spatial separation of electrons and holes. Moreover, the exchange interaction is also influenced by the center-of-mass momentum of excitons[24,25]. Therefore, both temperature and exciton density can effectively modulate the exchange interaction by altering the momentum distribution.

To quantitatively investigate these effects, we carried out magnetic field-dependent DCP measurements with different powers and temperatures. Representative results acquired at 4 K and 10 μW are summarized in **Fig. S18**. The **Fig. S19** and **Fig.**

S20 summarize the results of power- and temperature-dependent exchange interaction.

As shown in **Fig. S18**, we collected σ^- and σ^+ circularly polarized PL spectra under σ^- and σ^+ excitation, respectively (**Fig. S18a** and **Fig. S18b**). By fitting the PL intensities of IX and IXX, we can calculated their DCP using the formula:

$$DCP(\sigma^- \text{ exc}) = \frac{I(\sigma^-) - I(\sigma^+)}{I(\sigma^-) + I(\sigma^+)} \tag{S6}$$

$$DCP(\sigma^+ \text{ exc}) = \frac{I(\sigma^+) - I(\sigma^-)}{I(\sigma^+) + I(\sigma^-)} \tag{S7}$$

where $I(\sigma^+)$ and $I(\sigma^-)$ denote the fitted σ^+ and σ^- polarized PL intensity, respectively. As shown in **Fig. S18c** and **Fig. S18d**, both IX and IXX exhibit weak valley polarization at zero magnetic field. Under an out-of-plane magnetic field, their DCP rises rapidly and saturates at about 2 T.

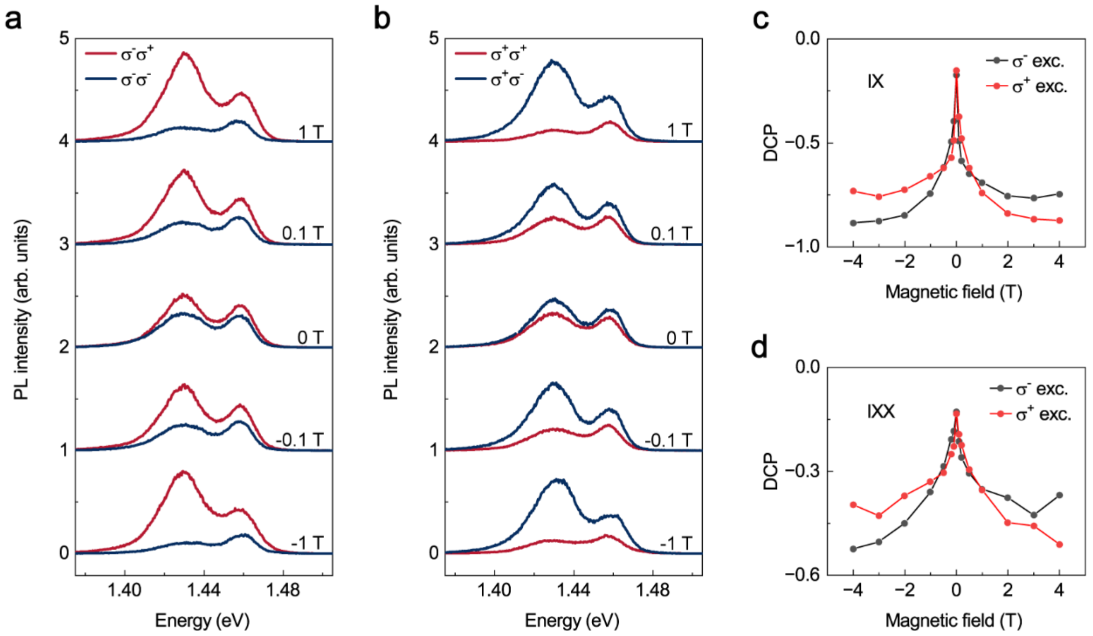

Fig. S18 Magnetic field dependent of DCP for IX and IXX at 4 K. a and **b** Circularly polarized PL spectra under σ^- (**a**) and σ^+ (**b**) excitation at different magnetic fields, with an excitation power of 10 μW. **c** and **d** DCP of IX (**c**) and IXX (**d**) under different magnetic fields.

To further analyze the valley polarization, we applied the method to define the valley polarization[25,26] (VP) as $VP = [DCP(\sigma^- \text{ exc}) + DCP(\sigma^+ \text{ exc})]/2$. The resulting VP as a function of magnetic field under different excitation powers is shown

in **Fig. S19a** and **Fig. S19b**. We fitted these data using the following model[25]:

$$VP = \frac{P_0}{1 + 2\frac{\tau}{\tau_{v0}}/[1 + (\frac{B}{B_c})^2]} \qquad (S8)$$

Here, τ_{v0} represents the valley lifetime at 0 field, τ is the exciton lifetime. B_c is the characteristic magnetic field that reflects the strength of the exchange interaction that reflects the strength of the exchange interaction.

As previous report[14], B_c is directly related to the exchange interaction strength via $J_{ex} \sim g\mu_B B_c$. We extracted B_c for both IX and IXX across different powers (**Fig. S19c**) and derived the corresponding J_{ex} values (**Fig. S19d**). For IX, J_{ex} increases from $0.06\ \text{meV}$ to $0.22\ \text{meV}$ with increasing power. This trend can be attributed to the enhancement of the center-of-mass momentum under higher exciton densities, which in turn leads to an enhancement of the exchange interaction. The exchange interaction strength in our work is also close to the $0.24\ \text{meV}$ reported for H-type heterostructures[14]. In contrast, the exchange interaction of IXX remains nearly constant over the same power range, indicating that it does not govern the DCP variation of the biexciton.

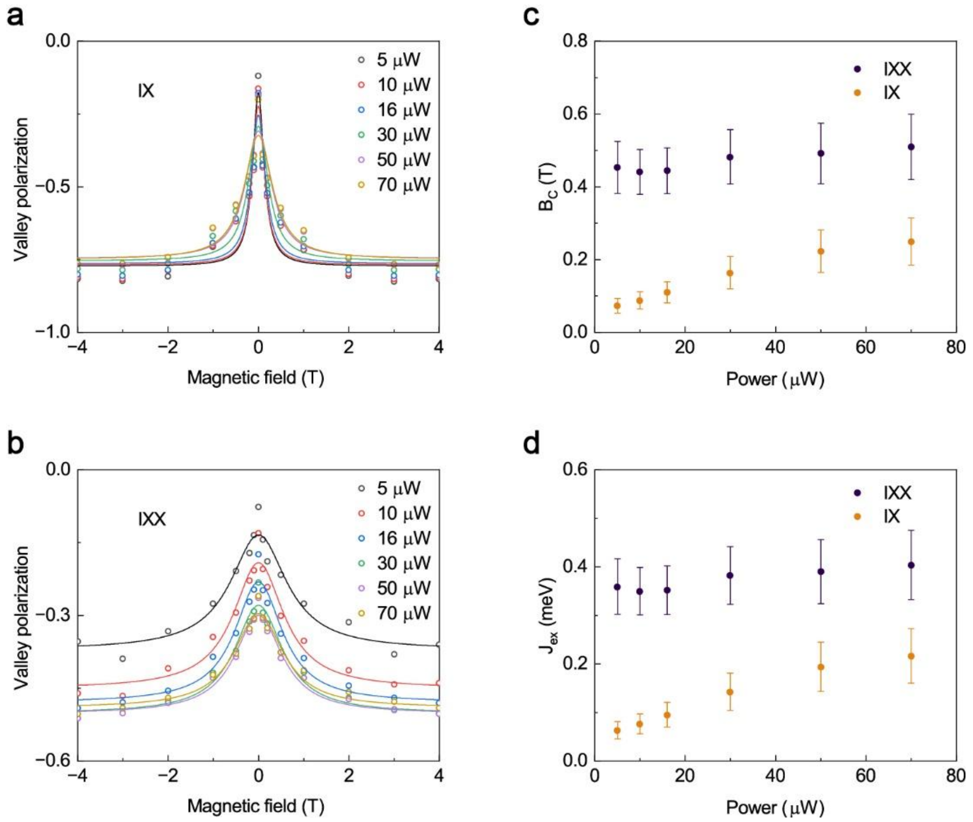

Fig. S19 Exchange interaction measurement at different excitation power. a and **b** Magnetic field dependence of valley polarization of IX (**a**) and IXX (**b**) at different excitation powers. Hollow circles represent data points, while the solid curve represents the result of fitting the data using the formula. **c** The characteristic magnetic fields B_c of IX and IXX at different powers. The error bars represent the fitting uncertainty of the characteristic magnetic field. **d** Exchange interaction strength of the IX and IXX at different powers. The error bars are obtained by the fitting uncertainty of the characteristic magnetic field. As the excitation power increases, the exchange interaction strength of IX increases, while the exchange interaction of IXX remains essentially unchanged.

Then we performed the similar analysis on temperature-dependent measurements at a fixed power of $16\ \mu\mathrm{W}$. The temperature-dependent valley polarization of IX and IXX is shown in **Fig. S20a** and **Fig. S20b**, and the extracted characteristic magnetic field B_c and exchange interaction J_ex are summarized in **Fig. S20c** and **Fig. S20d** at different temperatures, respectively. As the temperature increases, the exchange interaction strength of IX increases slightly, which is also consistent with the temperature induced increase of the center-of-mass momentum of IX. For the IXX, the large uncertainty in the IXX interaction strength makes it difficult to determine whether the trend decreases or remains constant with temperature. However, the increase in temperature maybe lead to an increase in the center-of-mass momentum, thus ruling out the possibility of a reduced exchange interaction strength. Therefore, we conclude that the IXX exchange interaction does not change with temperature.

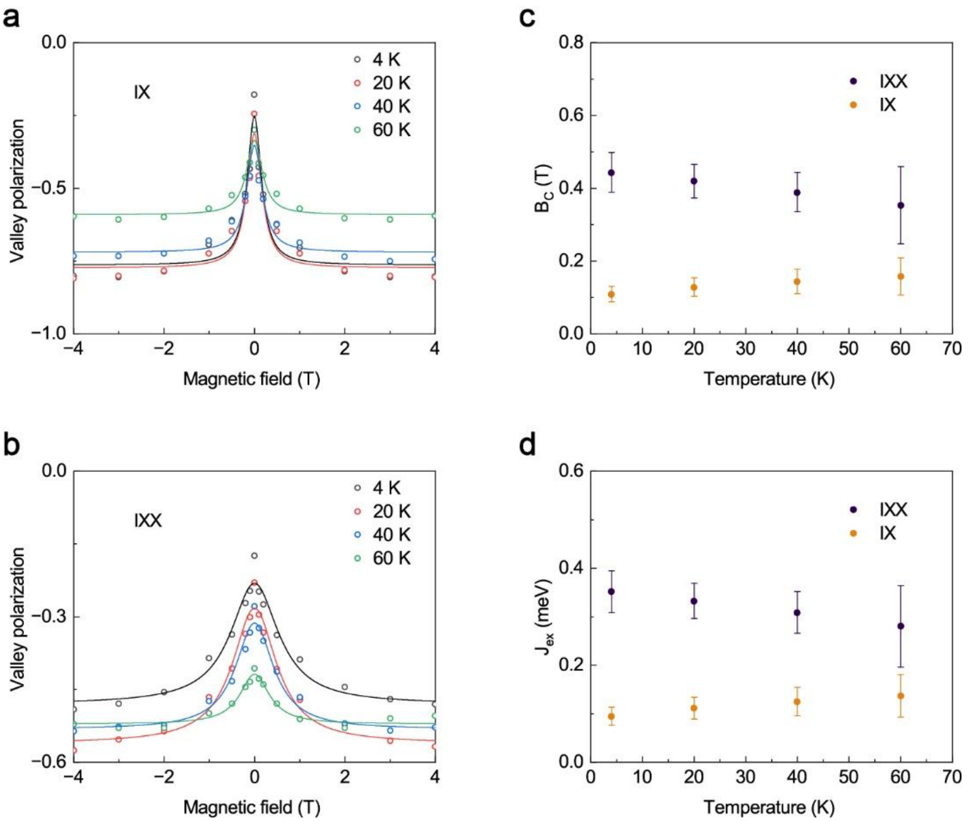

Fig. S20 Exchange interaction measurement at different temperatures and a power of 16 μW. a and **b** Magnetic field dependence of valley polarization of IX (**a**) and IXX (**b**) at different temperatures. Hollow circles represent data points, while the solid curve represents the result of fitting the data using the formula. **c** The characteristic magnetic fields B_c of IX and IXX at different temperatures. **d** Exchange interaction strength of IX and IXX at different temperatures. As the excitation power increases, the exchange interaction strength of IX increases. while the exchange interaction of IXX remains essentially unchanged.

Furthermore, due to strong Coulomb repulsion, the two constituent excitons of the IXX naturally have the larger center-of-mass momentum, hence resulting in a stronger exchange interaction for the IXX than that for the IX. This enhanced exchange interaction may promote the thermal equilibrium population of IXX between its two energy levels.

Supplementary Section 16: The peak position of interlayer exciton at different temperatures

Figure S21 shows interlayer exciton PL at different temperatures. Due to suppressed electron-phonon coupling at low temperatures[27,28], only a small redshift of approximately 2 meV is observed in the low-temperature emission peak from 4 K to 120 K (**Fig. S21a**). At higher temperatures, electron-phonon coupling is reactivated, resulting in a significant redshift of about 69 meV from 120 K to room temperature (**Fig. S21b**), consistent with reports in other literature[29].

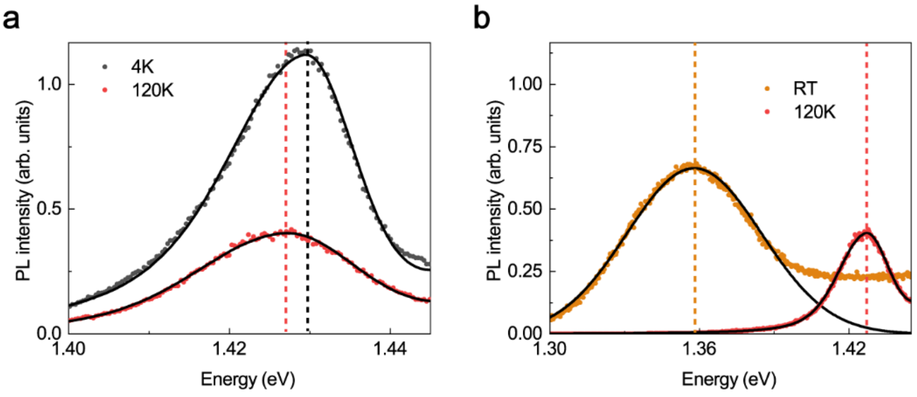

Fig. S21 Comparison of peak positions at different temperature. a The photoluminescence of interlayer exciton at 4 K and 120 K. The IX peak position is 1.429 meV at 4 K and 1.427 meV at 120 K, indicating a redshift of 2 meV. Black and red dashed lines mark the peak energy of 4 K and 120 K. **b** Comparison of photoluminescence at room temperature and 120 K. The deviation from a single-peak emission at the high-energy side originates from the broadening of intralayer excitons. The emission peak position at room temperature is 1.358 meV, which is redshifted by approximately 69 meV relative to the peak position at 120 K.

Supplementary Section 17: The power-dependent polarization of the IX and IXX at 20 K, 50 K and 80 K

Fig. S22 shows the power-dependent polarization of biexciton at different temperatures. Due to the enhanced nonradiative recombination at elevated temperatures, higher excitation powers is required to accurately capture the IXX PL intensity and the degree

of valley polarization (the gray arrow). At higher temperatures, we observe that the polarization of the IXX at low excitation powers also increases. Additionally, we note that there is a plateau of polarization at low powers, where the polarization of IXX changes slowly as power increases. The selected power in the **Fig. 4b** lies within this plateau.

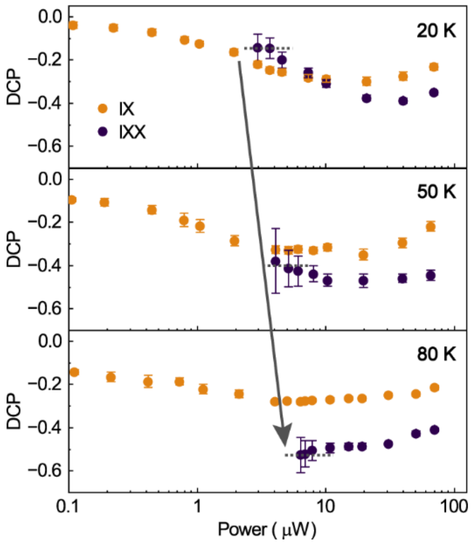

Fig. S22 The power-dependent polarization of the IX and IXX. The gray dotted line represents the plateau, where the polarization of IXX changes slowly with increasing power.

Supplementary Section 18: The model for temperature-dependent polarization of the IXX

We assume that the biexciton of X_+X_- and X_-X_- satisfy the thermal-activation function:

$$n_{X+X-}/n_{X-X-} \propto e^{\Delta_{IXX}/k_B T} \tag{S9}$$

Δ_{IXX} is the fine structure splitting between X_+X_- and X_-X_-. If the relaxation process is ignored, we can estimate the biexciton valley polarization:

$$DCP = -\frac{(I_{X+X-}+2I_{X-X-})-I_{X+X-}}{2I_{X-X-}+2I_{X+X-}} \tag{S10}$$

where the minus sign represents the opposite selection rule for interlayer exciton. I_{X-X-} and I_{X+X-} represent PL intensity of X_-X_- and X_+X_-, which is related to exciton

density and lifetime:

$$I_{X-X-} \propto n_{X-X-}/\tau_{X-X-}$$

$$I_{X+X-} \propto n_{X+X-}/\tau_{X+X-} \quad \text{(S11)}$$

Thus, we add (S9) and (S11) plug into (S10), temperature-dependent polarization of IXX is given by

$$DCP = -\frac{1}{1+\Gamma e^{\Delta_{IXX}/k_B T}} \quad \text{(S12)}$$

From fitting temperature-dependent polarization shown in **Figure 4b**, we get $\Delta_{IXX} = 2.77 \pm 0.19$ meV. Small fine structures splitting is the core of regulating polarization by temperature.

Another fitting parameter $\Gamma = 0.67 \pm 0.04$, which maybe be related to several factors that influence the biexciton. One possibility is that X_-X_- and X_+X_- have different rates of radiative recombination, which could lead to variations in their PL intensities, as described in formula (S11). Additionally, since biexciton are excited in moiré sites occupied by single exciton, thus differing exciton density of X_+ and X_- play the role of the distinct "density of states" for X_+X_- and X_-X_-. At low temperatures, the distribution of X_+X_- is higher than that of X_-X_-. Since the power we selected is enough small, the moiré sites occupied by X_+ are not full, so the effect of the density of states can be neglected[30]. However, as the temperature increases, $e^{\Delta_{IXX}/K_B T}$ approaches 1. Since both X_+ and X_- are present simultaneously, the density of states may have an impact, and the polarization of IXX will be relatively large.

Supplementary Section 19: The theoretical Calculation of the biexciton fine structure

For a single exciton within a moiré potential, the potential energy term can be approximated using a harmonic oscillator model [17]:

$$H_{IX}(\mathbf{r})\Phi^{(IX)} = E^{(IX)}\Phi^{(IX)}$$

$$H_{IX}(\mathbf{r}) = -\frac{\hbar^2}{2M}\Delta_{\mathbf{r}} + \frac{1}{2}M\omega_0^2|\mathbf{r}|^2 \quad \text{(S13)}$$

Here $\mathbf{r}$ is the coordinates of the exciton, M is the exciton mass. Within the parabolic approximation, the eigenstates and eigenenergy can be obtained analytically:

$$\Phi_{n,l}^{(\mathrm{IX})}(\mathbf{r},\theta)=\sqrt{\frac{n!}{\pi(n+|l|)!}}\frac{\mathbf{r}^{|l|}}{a^{|l|+1}}\mathcal{L}_n^{|l|}\left(\frac{\mathbf{r}^2}{a^2}\right)e^{-\frac{\mathbf{r}^2}{2a^2}+il\theta} \quad (S14)$$

$$E_{n,l}^{(\mathrm{IX})}=\hbar\omega_0(2n+|l|+1) \quad (S15)$$

Where n and l represent the radial quantum number and angular momentum quantum number, $n,|l|=0,1,2,\dots$. The factor $a^2=\frac{\hbar}{M\omega_0}$. The $\mathcal{L}_n^{|l|}\left(\frac{\mathbf{r}^2}{a^2}\right)$ represent Laguerre polynomials.

Next, we focus on the biexciton Hamiltonian. Interlayer exciton exhibits permanent out-of-plane dipole moment due to the spatial separation of electron and hole. Therefore, considering the exciton-exciton Coulomb repulsion between two excitons[31], the biexciton Hamiltonian follows the formalism:

$$\big(H_{\mathrm{IX}}(\mathbf{r_1})+H_{\mathrm{IX}}(\mathbf{r_2})+V_{\mathrm{dd}}(\mathbf{r_1}-\mathbf{r_2})\big)\Phi^{(\mathrm{IXX})}(\mathbf{r_1},\mathbf{r_2})=E^{(\mathrm{IXX})}\Phi^{(\mathrm{IXX})}(\mathbf{r_1},\mathbf{r_2})$$

$$V_{\mathrm{dd}}(\mathbf{r_1}-\mathbf{r_2})=\frac{e^2}{4\pi\epsilon_r\epsilon_0}\left(\frac{2}{|\mathbf{r_1}-\mathbf{r_2}|}-\frac{2}{\sqrt{d^2+|\mathbf{r_1}-\mathbf{r_2}|^2}}\right) \quad (S16)$$

where $\mathbf{r_1}$ and $\mathbf{r_2}$ are the coordinates of the two excitons, respectively. For WS_2/WSe_2 heterostructures, interlayer distance $d=0.65\,nm$ and averaged dielectric constant $\epsilon_r=5$. Therefore, the Hamiltonian of biexciton with exciton-exciton Coulomb repulsion fulfil:

$$(-\frac{\hbar^2}{2(2M)}\Delta_{\mathbf{R}}-\frac{\hbar^2}{2\left(\frac{M}{2}\right)}\Delta_{\mathbf{r}}+\frac{1}{2}(2M)\omega_0^2|\mathbf{R}|^2+\frac{1}{2}\left(\frac{M}{2}\right)\omega_0^2|\mathbf{r}|^2+$$

$$V_{\mathrm{dd}}(\mathbf{r}))\Phi^{(\mathrm{IXX})}(\mathbf{R},\boldsymbol{r})=E^{(\mathrm{IXX})}\Phi^{(\mathrm{IXX})}(\mathbf{R},\mathbf{r}) \quad (S17)$$

where we have introduced relative ($\mathbf{r}$) and centre-of-coordinates ($\mathbf{R}$):

$$\mathbf{R}=\frac{\mathbf{r_1}+\mathbf{r_1}}{2} \quad (S18)$$

$$\mathbf{r}=\mathbf{r_1}-\mathbf{r_2} \quad (S19)$$

We can separate the relative and centre-of-coordinates, $\Phi^{(\mathrm{IXX})}(\mathbf{R},\mathbf{r})=\Phi^c(\mathbf{R})\cdot\Phi^r(\mathbf{r})$. Similar to the treatment for single exciton, we get Hamiltonian and solutions of the centre-of-coordinate:

$$\left(-\frac{\hbar^2}{2(2M)}\Delta_{\mathbf{R}}+\frac{1}{2}(2M)\omega_0^2|\mathbf{R}|^2\right)\Phi^c(\mathbf{R})=E^c\Phi^c(\mathbf{R}) \tag{S20}$$

$$\Phi^c(\mathbf{R})=\Phi^{(\mathrm{IX})}_{n_R,l_R}\,(R,\theta) \tag{S21}$$

$$E^{(c)}_{n_R,l_R}\ =\hbar\omega_0(2n_c+|l_c|+1) \tag{S22}$$

Where n_R and l_R represent the radial quantum number and angular momentum quantum number. For the relative motion, the Hamiltonian equation is no longer a harmonic oscillator equation:

$$\left(-\frac{\hbar^2}{2\left(\frac{M}{2}\right)}\Delta_{\mathbf{r}}+\frac{1}{2}\left(\frac{M}{2}\right)\omega_0^2|\mathbf{r}|^2+V_{\mathrm{dd}}(\mathbf{r})\right)\Phi^{(r)}(\mathbf{r})=E^{(r)}\Phi^{(r)}(\mathbf{r}) \tag{S23}$$

The total potential is given by the moiré potential and Coulomb interaction. We can solve the corresponding eigenvalue problem by expanding the relative wave function in terms of the unperturbed harmonic oscillators with quantum numbers n_r and l_r. Given that the radial symmetry of the interaction potential forbids the mixing of different angular momentum states, so that l_r is still a good quantum number, and consequently, the eigenstates take the following form:

$$\Phi^{(r)}_{l_r}=\sum_{n_r=0}^{\infty}C_{n_r,l_r}\,\Phi^{(IX)}_{n_r,l_r}(r,\theta) \tag{S24}$$

where $\Phi^{(\mathrm{IX})}_{n_r,l_r}(r,\theta)$ is the wave function of the unperturbed harmonic oscillators with total mass $M/2$. The coefficients have to fulfil the equation:

$$\sum_{n_r'=0}^{\infty}(\hbar\omega_0(2n_r'+|l_r|+1)\delta_{n_r',n_r}+V_{n_r',n_r})C_{n_r',l_r}=E^{(r)}_{l_r}C_{n_r,l_r} \tag{S25}$$

Before calculating the energy of biexciton numerically, it is necessary to discuss the symmetry of the wave function. As established in (S18) and (S19), exchanging the two excitons $\mathbf{r_1}$ and $\mathbf{r_2}$ leaves the center-of-mass coordinate invariant but reverses the relative coordinate direction, such that $\mathbf{r}\rightarrow-\mathbf{r}$. Consequently, in (S14) and (S24), the angular coordinate transforms as $\theta_r\rightarrow\theta_r+\pi$. This transformation introduces a phase factor $e^{il_r\pi}=(-1)^{l_r}$. Therefore, the states with odd $|l_r|$ are antisymmetric under particle exchange, while states with even $|l_r|$ are symmetric.

For intravalley biexcitons, the valley pseudospin $|\uparrow\uparrow\rangle$ or $|\downarrow\downarrow\rangle$, requires a symmetric spatial wave function under bosonic exchange. For intervalley biexcitons, the valley pseudospin may be either the antisymmetric singlet $\frac{|\uparrow\downarrow\rangle-|\downarrow\uparrow\rangle}{2}$ or symmetric triplet $\frac{|\uparrow\downarrow\rangle+|\downarrow\uparrow\rangle}{2}$, resulting in antisymmetric spatial wave functions for singlet state or symmetric spatial wave functions for triplet state, respectively. We designate these as singlet (spatially antisymmetric) and triplet (spatially symmetric) states, which exhibit distinct angular momentum quantum numbers l_r : odd $|l_r|$ for singlet state and even $|l_r|$ for triplet state.

Now we calculate biexciton energy, which is the sum of centre-of-coordinate energy $E^{(c)}_{n_R,l_R}$ and relative motion energy $E^{(r)}_{l_r}$, expressed by:

$$E_{\mathrm{IXX}} = E^{(c)}_{n_R,l_R} + E^{(r)}_{l_r} \qquad (S26)$$

For the centre-of-coordinates $\mathbf{R}$, there are two quantum numbers, n_R and l_R, in the wave function. As these increase, the energies of the triplet and singlet state increase together. Therefore, we only calculate the energy of the ground state, i.e., $n_R = 0$ and $l_R = 0$. In this case, from equation (S22), we can obtain $E^{(c)}_{n_R=0,l_R=0} = \hbar\omega_0$.

For the relative motion $\mathbf{r}$, $E^{(r)}_{l_r}$ can be calculated theoretically based on the (S25) iterating over all n_r. However, it is found that as n_r increases, the energy $E^{(r)}_{l_r}$ rises significantly leading to the very weak hybridization between the high-energy and low-energy states. Consequently, as a good approximation, we ignore the high energy states and solve the hybridization problem considering only the states with $n_r = 0$ and $n_r = 1$. In this case, the wave function in (S24) becomes only dependent of the quantum number l_r. Although the biexciton energy increases with increasing l_r, here we are just interested the ground state eigenfunctions and eigenenergies of moiré potential trapped intervalley biexciton (singlet, $l_r = \pm 1$) and intravalley biexciton (triplet, $l_r = 0$) states. Based on above discussion, according to (S26), the energy of the biexciton is equal to $E^{(r)}_{l_r} + \hbar\omega_0$ when $E^{(c)}_{n_R=0,l_R=0} = \hbar\omega_0$.

Note that the above biexciton energy includes the characteristic energy $\hbar\omega_0$,

which can be derived from $U_0 \sim \frac{1}{2} M \omega_0{}^2 (\frac{r_m}{2})^2$, where $U_0 = 90\ \mathrm{meV}$ and $r_m = 7.6\ \mathrm{nm}$ is moiré potential and moiré period[1,30], M is exciton effective masses in heterostructure. It has been pointed that the band renormalization of heterostructures yields M increasing significantly. For example, in near-0° twisted WS_2/WSe_2, the hole mass is approximately $3 \sim 8\ m_0$ [32]. Given comparable electron and hole effective masses[33], we estimate the exciton mass as $M = 10\ m_0$. This aligns with prediction of $7\ m_0$ at 1° twist angles[34], and we anticipate an even larger effective mass in 59.8° twisted WS_2/WSe_2 heterostructure due to enhanced moiré confinement. Substituting the values, we obtain theoretical value $\hbar\omega_0 \sim 9.8\ \mathrm{meV}$.

In order to understand how the valley pseudospin modulate the spatial wave functions as well as the related energy, we solved (S25) and obtained the parameters C_{n_r, l_r} for triplet state and singlet state. Substituting these parameters into (S24), we obtained the wave function $\Phi^{(r)}_{l_r=0}$ and $\Phi^{(r)}_{|l_r|=1}$, the probability density $|\Phi^{(r)}_{l_r=0}|^2$ for triplet state and $|\Phi^{(r)}_{|l_r|=1}|^2$ for singlet state are shown in the **Fig.4d** and **Fig.4e**. As seen, the triplet state exhibits a smaller average exciton distance and a peak probability density at $r = 1.77\ \mathrm{nm}$, while the singlet state displays a larger average exciton distance and peaks at $r = 2.16\ \mathrm{nm}$. Therefore, we infer that the triplet state experiences stronger Coulomb interactions and consequently possesses higher energy.

It is known from (S16) that the biexciton energy is divided into two parts $E_0 + U$. the first term is the ground state energy $E_0 = \left\langle \Phi^{(r)}_{l_r} \middle| H_{\mathrm{IX}}(\boldsymbol{r_1}) + H_{\mathrm{IX}}(\boldsymbol{r_2}) \middle| \Phi^{(r)}_{l_r} \right\rangle$, which corresponds to the harmonic oscillator energy of the two-exciton system. The second term is the Coulomb repulsion $U = \left\langle \Phi^{(r)}_{l_r} \middle| V_{\mathrm{dd}}(\boldsymbol{r_1} - \boldsymbol{r_2}) \middle| \Phi^{(r)}_{l_r} \right\rangle$. This allows us to compute these contributions. Moreover, solving equation (S25) directly yields the energy $E^{(r)}_{l_r}$, and enabling determination of biexciton energies on triplet state ($|l_r| = 0$) and singlet state ($|l_r| = 1$), respectively. **Fig. S23** shows the biexcitons energies relative to the single exciton (the energy of a single exciton set to zero). It can be found that, owing to the non-zero angular momentum, the singlet state has a higher ground state

energy $E_0^{\mathrm{s}} = 17.2\,\mathrm{meV}$ (blue line) than that of the triplet state exhibits $E_0^{\mathrm{t}} = 10.8\,\mathrm{meV}$ (red line), when $\hbar\omega_0 = 9.8\,\mathrm{meV}$. However, after including the Coulomb repulsion interaction energy, the energy of triplet state (orange line) surpasses that of the singlet state, this is in line with our previous expectations.

Lastly, it is worth pointing out that two important calculated data can be deduced from **Fig. S23**. One is the average biexciton energy relative to single exciton being around $30\,\mathrm{meV}$, this is very consistent with the experimental observation shown in **Fig.1** in main text. The other is that the singlet-triplet energy splitting is about $2.7\,\mathrm{meV}$, which is excellent agreement with the fine structure between the intravalley and intervalley biexcitons experimental finding in **Fig. 3** and **Fig. 4**.

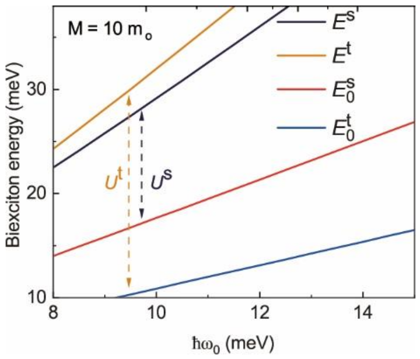

Fig. S23 the ground state energy (E_0^{t}, E_0^{s}) and the biexciton energy (E^{t}, E^{s}) for triplet and singlet state. U^{t} and U^{s} represent the Coulomb repulsion energy of triplet and singlet state. The lower ground state energy E_0^{t} of the triplet state counterbalances its enhanced Coulomb repulsion U^{t}, yielding a net energy splitting of $2.7\,\mathrm{meV}$ between triplet and singlet states.

References

1. Lian, Z. et al. Valley-polarized excitonic Mott insulator in WS_2/WSe_2 moiré superlattice. *Nat. Phys*. **20**, 34-39 (2024).
2. Park, H. et al. Dipole ladders with large Hubbard interaction in a moiré exciton lattice. *Nat. Phys*. **19**, 1286-1292 (2023).
3. Tang, Y. et al. Simulation of Hubbard model physics in WSe_2/WS_2 moiré superlattices. *Nature*. **579**, 353-358 (2020).
4. Jadczak, J. et al. Room temperature multi-phonon upconversion photoluminescence in monolayer semiconductor WS_2. *Nat. Commun.* **10**, 107 (2019).
5. Xu, Y. et al. Correlated insulating states at fractional fillings of moiré superlattices. *Nature*. **587**, 214-218 (2020).
6. Moody, G. et al. Intrinsic homogeneous linewidth and broadening mechanisms of excitons in monolayer transition metal dichalcogenides. *Nat. Commun.* **6**, 8315 (2015).
7. Tang, Y. et al. Tuning layer-hybridized moiré excitons by the quantum-confined Stark effect. *Nat. Nanotechnol.* **16**, 52-57 (2021).
8. Li, Z. et al. Revealing the biexciton and trion-exciton complexes in BN encapsulated WSe_2. *Nat. Commun.* **9**, 3719 (2018).
9. Deng, S. et al. Frozen non-equilibrium dynamics of exciton Mott insulators in moiré superlattices. *Nat. Mater*. **24**, 527-534 (2025).
10. Zhao, S. et al. Excitons in mesoscopically reconstructed moiré heterostructures. *Nat. Nanotechnol.* **18**, 572-579 (2023).
11. Yu, H., Liu, G. & Yao, W. Brightened spin-triplet interlayer excitons and optical selection rules in van der Waals heterobilayers. *2D Mater*. **5**, 35021 (2018).
12. Baek, H. et al. Highly energy-tunable quantum light from moire-trapped excitons. *Sci. Adv*. **6**, 8526-8537 (2020).
13. Regan, E. C. et al. Emerging exciton physics in transition metal dichalcogenide heterobilayers. *Nature Reviews Materials*. **7**, 778-795 (2022).

14. Wu, Y. et al. Highly tunable valley polarization of potential-trapped moire excitons in WSe_2/WS_2 Heterojunctions. *Phys. Rev. Lett.* **134**, 256402 (2025).
15. Lian, Z. et al. Valley-polarized excitonic Mott insulator in WS2/WSe2 moiré superlattice. *Nat. Phys.* **20**, 34-39 (2024).
16. Fang, H. et al. Localization and interaction of interlayer excitons in $MoSe_2/WSe_2$ heterobilayers. *Nat. Commun*. **14**, 6910 (2023).
17. Brem, S. & Malic, E. Optical signatures of moiré trapped biexcitons. *2D Mater*. **11**, 25030 (2024).
18. Cai, C. et al. Ultralow auger-assisted interlayer exciton annihilation in WS_2/WSe_2 moiré heterobilayers. *Nano Lett.* **24**, 2773-2781 (2024).
19. Li, W. J., Lu, X., Dubey, S., Devenica, L. & Srivastava, A. Dipolar interactions between localized interlayer excitons in van der Waals heterostructures. *Nat. Mater*. **19**, 624-629 (2020).
20. Tan, Q., Rasmita, A., Zhang, Z., Novoselov, K. S. & Gao, W. Signature of cascade transitions between interlayer excitons in a moiré superlattice. *Phys. Rev. Lett*. **129**, 247401 (2022).
21. Tran, K. et al. Evidence for moiré excitons in van der Waals heterostructures. *Nature*. **567**, 71-75 (2019).
22. Li, W., Lu, X., Wu, J. & Srivastava, A. Optical control of the valley Zeeman effect through many-exciton interactions. *Nat. Nanotechnol.* **16**, 148-152 (2021).
23. Steinhoff, A. et al. Exciton-exciton interactions in van der Waals heterobilayers. *Phys. Rev. X.* **14**, 31025 (2024).
24. Yu, T. & Wu, M. W. Valley depolarization due to intervalley and intravalley electron-hole exchange interactions in monolayer MoS_2. *Phys. Rev. B*. **89**, 205303 (2014).
25. She, Y. et al. Magneto-Polarization Controlled by Intervalley Scattering of Interlayer Excitons and Carriers in WS_2/WSe_2 Heterostructure. *Nano Lett.* **25**, 6708-6715 (2025).
26. Jiang, C. et al. Microsecond dark-exciton valley polarization memory in two-

dimensional heterostructures. *Nat. Commun.* **9**, 753 (2018).

27. Antonius, G. & Louie, S. G. Theory of exciton-phonon coupling. *Phys. Rev. B*. **105**, 85111 (2022).
28. Wang, X. H. et al. Influence of lattice vibrations on luminescence and transfer of excitons in WS_2 monolayer semiconductors. *J. Phys. D Appl. Phys.* **49**, 465101 (2016).
29. Ugeda, M. M. et al. Giant bandgap renormalization and excitonic effects in a monolayer transition metal dichalcogenide semiconductor. *Nat. Mater.* **13**, 1091-1095 (2014).
30. Xiong, R. et al. Tunable exciton valley-pseudospin orders in moiré superlattices. *Nat. Commun*. **15**, 4254 (2024).
31. Takahashi, S. et al. 3D hydrogen-like screening effect on excitons in hBN-encapsulated monolayer transition metal dichalcogenides. *Sci. Rep*. **14**, 27286 (2024).
32. Stansbury, C. H. et al. Visualizing electron localization of WS_2/WSe_2 moiré superlattices in momentum space. *Sci. Adv*. **7**, eabf4387 (2021).
33. Kormányos, A. et al. k p theory for two-dimensional transition metal dichalcogenide semiconductors. *2D Mater*. **2**, 22001 (2015).
34. Knorr, W., Brem, S., Meneghini, G. & Malic, E. Exciton transport in a moiré potential: From hopping to dispersive regime. *Phys. Rev. Mater*. **6**, 124002 (2022).
